# Atorvastatin Restores PPARα Inhibition of Lipid Metabolism Disorders by Downregulating miR-21 Expression to Improve Mitochondrial Function and Alleviate Diabetic Nephropathy Progression

**DOI:** 10.3389/fphar.2022.819787

**Published:** 2022-02-11

**Authors:** Jiayi Xiang, Huifang Zhang, Xingcheng Zhou, Dan Wang, Rongyu Chen, Wanlin Tan, Luqun Liang, Mingjun Shi, Fan Zhang, Ying Xiao, Yuxia Zhou, Yuanyuan Wang, Bing Guo

**Affiliations:** ^1^ State Key Laboratory of Functions and Applications of Medicinal Plants, Guizhou Medical University, Guiyang, China; ^2^ Department of Pathophysiology, Guizhou Medical University, Guizhou, China; ^3^ International Scientific and Technological Cooperation Base of Pathogenesis and Drug Research on Common Major Diseases, Guizhou Medical University, Guizhou, China

**Keywords:** atorvastatin, miR-21, PPARα, fenofibrate, mitochondrial dynamics, diabetic kidney disease, lipid metabolism disorders

## Abstract

Atorvastatin is a classical lipid-lowering drug. It has been reported to have renoprotective effects, such as reducing urinary protein excretion and extracellular matrix aggregation. The present study aimed to investigate the specific mechanism of action of Atorvastatin in type 1 diabetic mice (T1DM) in inhibiting renal tubular epithelial cell injury following treatment with high glucose and high fat. The anti-injury mechanism of Atorvastatin involved the inhibition of miR-21 expression and the upregulation of the transcription and expression of its downstream gene Peroxisome proliferator-activated receptors-α(PPARα). An increase in blood glucose and lipid levels was noted in the T1DM model, which was associated with renal fibrosis and inflammation. These changes were accompanied by increased miR-21 levels, downregulation of PPARα and Mfn1 expressions, and upregulation of Drp1 and IL6 expressions in renal tissues. These phenomena were reversed following the administration of Atorvastatin. miR-21 targeted PPARα by inhibiting its mRNA translation. Inhibition of miR-21 expression or Fenofibrate (PPARα agonist) administration prevented the decrease of PPARα in renal tubular epithelial cells under high glucose (HG) and high fat (Palmitic acid, PA) conditions, alleviating lipid metabolism disorders and reducing mitochondrial dynamics and inflammation. Consistent with the *in vivo* results, the *in vitro* findings also demonstrated that mRTECs administered with Atorvastatin in HG + PA increased PPARα expression and restored the normal expression of Mfn1 and Drp1, and effectively increasing the number of biologically active mitochondria and ATP content, reducing ROS production, and restoring mitochondrial membrane potential following Atorvastatin intervention. In addition, these effects were noted to the inhibition of FN expression and tubular cell inflammatory response; however, in the presence of miR-21mimics, the aforementioned effects of Atorvastatin were significantly diminished. Based on these observations, we conclude that Atorvastatin inhibits tubular epithelial cell injury in T1DM with concomitant induction of lipid metabolism disorders by a mechanism involving inhibition of miR-21 expression and consequent upregulation of PPARα expression. Moreover, Atorvastatin regulated lipid metabolism homeostasis and PPARα to restore mitochondrial function. The results emphasize the potential of Atorvastatin to exhibit lipid-regulating functions and non-lipid effects that balance mitochondrial dynamics.

## Introduction

In 2019, the International Diabetes Federation (IDF) predicted that the global prevalence of diabetes will be 9.3% (463 million people). This incidence is predicted to rise to 10.2% (578 million people) by 2030 and to 10.9% (700 million people) by 2045. As one of the most common microvascular complications of diabetes, diabetic nephropathy (DN) is a major cause of the end-stage renal disease (ESRD) (Saeedi et al., 2019). Diabetic kidney disease (DKD) has been a difficult clinical problem to treat, and intensive glycemic control can only reduce but not eradicate the progression of the disease. It has been found that patients with diabetes mellitus usually have abnormal glucose metabolism along with abnormal lipid metabolism. Dyslipidemia is a significant risk factor for the development of DN (Herman-Edelstein et al., 2014).

As a highly perfused organ, the renal tubules are the main site of transmembrane transport of substances in the kidney, and therefore abundant mitochondria are present in the tubular epithelial cells (Bhargava and Schnellmann, 2017). The participation of oxygen allows mitochondria to synthesize large amounts of ATP through oxidative phosphorylation to ensure the energy supply of the renal tubules. Mitochondrial dynamics is the process of determining the length, shape, and size of mitochondria. Mitochondria are dynamic organelles that adapt to cellular energy demands through morphological changes. Mitochondria regulate their morphology through fusion and fission, which is further regulated mainly by the balance between mitochondrial fission and fusion factors. These factors are essential for the repair of damaged mitochondrial components, allowing the exchange of materials between damaged and undamaged mitochondria through the fusion process, or the separation of damaged components through the fission process. This process maintains appropriate mitochondrial dynamics, which is essential for their normal function (Chen and Chan, 2004; Jheng et al., 2012). Mitochondria are also one of the main sources of the production of cellular ROS. Damaged mitochondria can substantially increase ROS production, leading to oxidative stress and tissue damage (Yu et al., 2006; Gerber and Rutter, 2017). Persistent mitochondrial dysfunction plays an important role in the progression of renal diseases, such as acute kidney injury (AKI) and diabetic nephropathy. It has been reported that hyperglycemia increases the production of NADH and FADH2 through the tricarboxylic acid cycle, while it promotes the mitochondrial electron transport chain (ETC), and releases ROS, which impairs and blocks the expression of proteins that affect mitochondrial function (Simon and Hertig, 2015; Coughlan and Sharma, 2016). This ultimately leads to the development of DKD. Therefore, improving mitochondrial dynamics and restoring their function has the potential to restore renal function.

Statins are a class of clinical hypolipidemic agents that reduce cholesterol biosynthesis by inhibiting the activity of 3-hydroxy-3-methyl-Glu-Daryl (HMG)-CoA reductase 6. This results in lowering lipid levels (Kogawa et al., 2019). A clinical study (Athyros et al., 2015) indicated that statin therapy reduced the rate of proteinuria and renal function loss. Recently, several studies have shown that statins can regulate the expression of miRNAs. For example, Atorvastatin treatment reduced the expression levels of miR-29b, miR-214, and miR-36-3p in human umbilical vascular endothelial cells after the establishment of an *in vitro* atherosclerotic cell model (Jia et al., 2019). Atorvastatin also downregulated miR-21 expression in a rat model of bile duct ligation (BDL)-induced liver fibrosis (Nozari et al., 2020). Furthermore, one study demonstrated that Atorvastatin protected the kidneys of diabetic rats by inhibiting the expression of inflammatory factors (Liao et al., 2016). Although accumulating evidence has demonstrated the potential benefit of statins in diabetic nephropathy, the exact mechanism has not been fully elucidated. In the present study, we investigated whether Atorvastatin was involved in delaying diabetic kidney fibrosis via inhibition of miR-21 expression.

Peroxisome proliferator-activated receptor-α (PPARα) is a ligand-dependent nuclear receptor that can be activated by exogenous compounds, such as fibrates, or by endogenous ligands, including fatty acids and prostaglandins. It is an important transcriptional regulator of genes involved in peroxisome and mitochondrial β-oxidation, FA transport, and hepatic gluconeogenesis, with important antioxidant, anti-inflammatory, and antiapoptotic roles (Xu et al., 2002). In addition, PPARα is involved in the activation of autophagy and mitochondrial homeostasis in mice infected with *Mycobacterium avium* via nuclear-mitochondrial interactions (Kim et al., 2019). It has been reported in the literature that miR-21 was found to inhibit the expression of PPARα in both cardiac tissue (Chuppa et al., 2018) and Hela cells (Zhou et al., 2011). The predicted results were analyzed by the bioinformatics website Targetscan and indicated that the seed sequence of miR-21 (5′-AGCUUA-'3) was complementary to the 3′UTR sequence of human PPARα mRNA (5′-UAAGCU-3′). It remains unclear whether miR-21 acts on PPARα and affects lipid metabolism disorders and mitochondrial dynamics, which are involved in the course of DKD.

In the present study, we demonstrated that Atorvastatin could inhibit DKD fibrosis by inhibiting miR-21 expression. Moreover, it was able to restore the levels of PPARα, which is a key transcription factor that regulates lipid metabolism, and improve mitochondrial dynamics.

## Materials and Methods

### Chemicals and Antibodies

The primary antibodies used against the proteins were as follows: Anti-actin (1:1,000), obtained from PumeiBiotechnology (Pumei, China); anti-fibronectin (1:1,000), anti-collagen-I (1:1,000), anti-α-smooth muscle actin (α-SMA, 1:1000), anti-interleukin 6 (IL-6,1:1000), anti-PPARα (1:1000 for Western blot, 1:100 for immunohistochemical staining), anti-CPT1a (1:1,000), anti-Mfn1 ((1:1,000 for Western blot, 1:100 for immunohistochemical staining) and anti-Drp1, which were obtained from ProteintechGroup (Proteintech, China);

### Animal Models

The T1DM model was established in C57 black mouse (4–6weeks, 18–22 g,males) (Liaoning Changsheng Biotechnology Co. Ltd., Liaoning, China). The animals were randomly divided into the normal control (NC) group (n = 6) and the DM group (n = 12). The mice of the DM group were intraperitoneally injected with 55 mg/kg streptozotocin (STZ, Sigma); the mice in the NC group were injected with the same amount of pH 4.5 sterile citric acid-sodium citrate buffer (lysozyme) for five consecutive days. Fasting blood glucose levels in mice were assessed at 72 h following treatment, and values ≥16.7 mmol/l indicated that DM mice were successfully established. Following 5 weeks of feeding, the diabetic mice were randomized into the diabetic group (n = 6) and Ato group (DM + Ato) (n = 6). Atorvastatin was administered at 20 mg/(kgd) (Pfizer, China) to the Ato group for 4 weeks. The NC and DM groups were intragastrically administered with carboxymethyl cellulose for 4 weeks. The mouse kidneys were collected at the ninth week. Urine samples were obtained and measured in the 24-h period preceding euthanasia. All mice had fasted 6 h prior to sacrifice. Blood specimens were collected from the femoral artery and centrifuged for serum preparation. The samples were kept at −20°C for biochemical assessment. Both kidneys were removed, of which one was stored at −80°C (RNA and protein preparations) and the other used for fixation with 4% formalin and subsequent histological and immunohistochemical evaluations. All animal studies complied with the regulations and guidelines of Guizhou Medical University institutional animal care and followed the AAALAC and IACUC guidelines. The approval form for Animal Experimentation Ethics Group of Guizhou Medical University certificate number (No. 1602230).

### Cell Culture and Transfection

Mouse renal tubular epithelial cells (mRTECs) were obtained from the Cell Bank of the Type Culture Collection, Shanghai Institute of Cell Biology, Chinese Academy of Sciences. The cells were maintained in Dulbecco’s modified Eagle’s medium (DMEM; Gibco, USA) supplemented with 10% fetal bovine serum (FBS; Gibco) and 5.5 mM glucose, in an incubator containing 5% CO_2_ at 37°C.

Cell proliferation was performed in the presence of normal glucose levels (NG; 5.5 mM), high glucose levels (HG; 30 mM), palmitic acid (PA; 0.2 mM), and high glucose levels with palmitic acid (HG 30 mM + PA 0.2 mM), which were supplemented with 2% FBS. mRTECs were transiently transfected with Lipofectamine 3,000 (Invitrogen, USA) based on the manufacture’s protocol. The relevant cell groups were treated with Atorvastatin (Ato; 10 μM; Pfizer, China) or Fenofibrate (a PPARα agonist; 25μM; APExBIO, USA). Si-PPARα was purchased from Longqian Biotech (China).

HK-2 cells were purchased from the American Type Culture Collection (ATCC^®^, Rockefeller, Maryland, USA) and cultured in Dulbecco’s modified Eagle medium/nutrient mixture F-12 (DMEM/F-12, Gibco, Grand Island, NY, USA) containing 10% fetal bovine serum (FBS, Gibco, USA) with 5% CO2 at 37°C. The cells in the logarithmic phase were used for subsequent experiments using the Luciferase reporter assay.

### Transfection of miR-21 Mimics or Inhibitor

miR-21 mimics, miR-21 inhibitor and their controls (Ribobio, China) were separately transfected into mRTECs and HK-2 cells. The procedure of transfection of miRNA mimics or inhibitors was performed as previously described.

### Biochemical Assays

Blood and urine specimens of mice were sent to Guiyang Jinwei (China) for detection of glucose, cholesterol, urea nitrogen, triglyceride, creatinine and 24 h urine microalbumin (mg/24 h). The 24 h urine microalbumin (mg/24 h) was assessed as follows: Microalbumin (mg/ml) × urine volume (ml)/24 h. The total Superoxide Dismutase (T-SOD) assay kit and the Malondialdehyde (MDA) assay kit of the mice were obtained from Nanjing Jiancheng Bioengineering Institute (Njjcbio, China). The assay was performed as described by the manufacturer.

### Histology and Immunohistochemical Staining

The paraffin sections of the kidney tissue samples were harvested. The resulting sections underwent staining with Hematoxylin-eosin (Solarbio, China), periodic acid-Schiff (Solarbio, China) and sirius red staining (BestBio, China) reagents according to corresponding recommended protocols. Diaminobenzidine (DAB) color developing kit (ZSGB-BIO, Beijing, China) and hematoxylin were used for immunohistochemical staining. The areas of positive staining were quantified by ImageJ in six random fields (200×) per sample, with three individuals assessed in each group.

### Western Blot Analysis

The kidney tissue and cell samples were lysed with RIPA buffer (R0020; Solarbio, China) and total protein amounts were determined with the BCA kit (PC0020; Solarbio). Following addition of the corresponding loading buffer (P1040 or P1019; Solarbio), the mixture was incubated for 10 min in boiling water. Equal amounts of total protein were resolved by SDS-PAGE and electro-transferred onto PVDF compound membranes (Millipore, USA) treated with methanol. Following blocking with 5% nonfat milk for 1 h at room temperature, the membranes were incubated with a primary antibody overnight at 4°C, and subsequently with horseradish peroxidase-conjugated secondary antibody for 1 h at room temperature. Finally, the ECL solution was added, and a Bio-Rad gel imaging system (Bio-Rad, USA) was employed for analysis.

### Real Time-Quantitative PCR

Total RNA was purified from kidneys and cells with TRIzol reagent (Invitrogen, USA) as described by the manufacturer. The Bulge-Loop™ miRNA qRT-PCR primer kit (Ribobio, China) was used to assess miR-21 expression levels. In addition, qPCR was carried out with SuperReal PreMix (SYBR Green) (Tiangen, China) and iQ SYBR Green SuperMix (Bio-Rad). The gene expression levels were normalized to those of GADPH or U6. The Bulge-Loop™ RT primer and qPCR primers specific for miR-21 and U6 genes were designed and synthesized by RiboBio (RiboBio, China). The 2−ΔΔCt method was employed for quantification. The sequences of the other primers used are described in Table 1.

**TABLE 1:** Primers used in qRT-PCR.

Gene	Sequence
PPARα-mouse	Forward: 5′-AAA​AGA​ATC​CCC​AGC​TTA​TCC​A-3′
	Reverse: 5′-TTG​GTG​ACT​TCC​CCT​AGG​TAT​A-3′
GAPDH-mouse	Forward: 5′-GAA​CGG​GAA​GCT​CAC​TGG-3′
	Reverse: 5′-GCC​TGC​TTC​ACC​ACC​TTC​T-3′
ACOX1-mouse	Forward: 5′-GGC​TTT​GGT​GGA​TGC​CTT​TG-3′
	Reverse: 5′-GGA​CTT​CTT​GCC​CAC​TCA​A-3′
CPT1a-mouse	Forward: 5′-CGG​CAG​ACC​TAT​TTT​GCA​CG-3′
	Reverse: 5′-TAG​ATG​CCT​CAG​GGT​CCT​CC-3′

The PPARα-promoter luciferase reporter was constructed by Longqian Biotech (China). Actively proliferating HK-2 cells were trypsinized and seeded in plates at a suitable density for routine culture. Following 24 h of incubation, transfection was carried out with Lipofectamine 3,000 (Invitrogen) as directed by the manufacturer for 48 h. This was followed by cell lysis and sample analysis with a Dual-Luciferase Reporter Assay System (E1960; Promega, USA). Renilla and Firefly luciferase activities were measured, and the ratio of Renilla luciferase activity to that of Firefly luciferase was derived. Triplicate experiments were independently repeated 3 times.

### Measurement of ATP Levels, ROS Production, Mitochondrial Membrane Potential, and Active Mitochondria

ATP was measured using an ATP Assay kit (Beyotime Biotechnology, China) as determined by the manufacturer’s instructions. The luminescence produced was measured with a luminometer counter (perkin-elmer, Waltham, MA and United States), and the concentration of ATP was calculated using an ATP standard curve.

Mitochondrial membrane and intracellular ROS were measured using the relevant assay kit (MedChemExpress,USA) as determined by the manufacturer’s instructions. Flow cytometry was performed on the NovoCyte Flow Cytometer (3,130; ACEA, China) and the data were analyzed by FlowJo software (Treestar, Ashland, OR, USA).

The active mitochondria in the primary cardiomyocytes were labeled with MitoTracker Red CMXRos probe (Beyotime Biotechnology, China) and imaged using a confocal laser-scanning microscope (Olympus + Confocal Microscope).

### Statistical Analysis

The assays were performed at least 3 times independently, and the animal experiments exhibited six samples per group. The data are indicative of mean ± standard deviation (SD). The unpaired Student t-test and the one-way analysis of variance (ANOVA) were carried out for group pair and multiple group comparisons, respectively. Spearman (nonparametric) correlation analysis was performed to evaluate the association of PPARα expression with miR-21. SPSS 22 was used for data analysis. *p* < 0.05 was considered to indicate statistical significant differences.

## Results

### Atorvastatin Improves Renal Fibrosis and Restores Renal Function in Diabetic Mice

**FIGURE 1:**
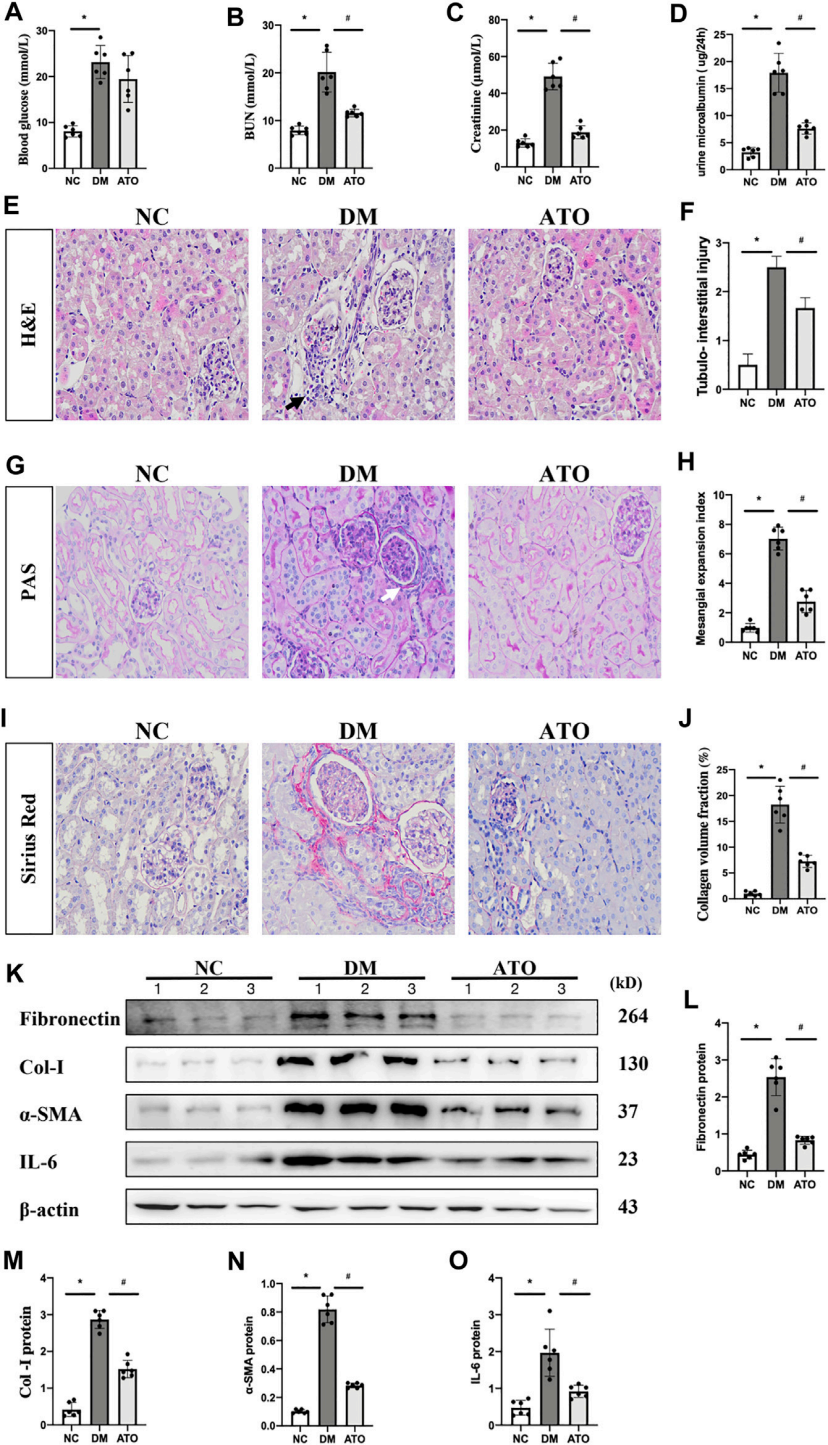
Atorvastatin improves renal fibrosis and restores renal function in diabetic mice. Effects of Atorvastatin on blood glucose, renal function, and fibrotic lesions in diabetic mice. **(A)** The blood glucose levels in the DM group were significantly higher than those in the control group, while no statistically significant difference was noted in the Atorvastatin group. The renal urea nitrogen **(B)**, serum creatinine **(C)**, and 24-h total urine microalbumin **(D)** levels were measured. H&E staining **(E)** was performed to observe the renal pathological changes in mice and to assess the tubular injury index **(F)**. PAS staining **(G)** was performed to assess the thylakoid expansion index **(H)**. Sirius red staining **(I)**, collagen deposition fraction **(J)**. Black arrows indicate the site of lymphocyte infiltration; white arrows indicate the glomerular basement membrane. Immunoblotting bands **(K)** and quantitative data **(L–O)** of FN, PPARα, Mfn1, Drp1, and IL-6 in each group of mice. All images are magnified ×200. NC: normal diet-fed rats; DM: type 1 diabetic mice; ATO: type 1 diabetic mice treated with Atorvastatin. All data are presented as mean ± SD from three independent experiments. n = 6; *p < 0.05 vs. NC group. #*p* < 0.05, compared with the DM group.

Diabetic mice indicated a significant increase in blood glucose levels and were characterized by a significant increase in renal function-related parameters (urea nitrogen, creatinine, and microalbuminuria) compared with age-matched non-diabetic control mice. The mice treated with Atorvastatin [20 mg/(kg-d)] for 4 weeks indicated no significant change in blood glucose levels (Figure 1A), whereas a significant decrease was noted in urea nitrogen and creatinine levels, as well as in the incidence of microalbuminuria (Figures 1B–D). H&E, PAS, and Sirius red staining indicated lymphocyte infiltration, thylakoid zone expansion, glomerular hypertrophy, tubular vacuole formation, and periglomerular fibrosis. In the Atorvastatin group, the lymphocyte infiltration was reduced, the thylakoid expansion fraction was decreased, and the fibrosis was significantly reduced (Figures 1E–J). The levels of fibronectin, collagen-I, α-SMA, and IL-6 were increased in the kidneys of DM mice compared with those of the control group. In contrast to these findings, the expression levels of the aforementioned proteins were decreased in the Atorvastatin group (Figures 1K–O).

### Atorvastatin Restores PPARα Expression and Improves Lipid Metabolism and Mitochondrial Dysfunction in Diabetic Mice

**FIGURE 2:**
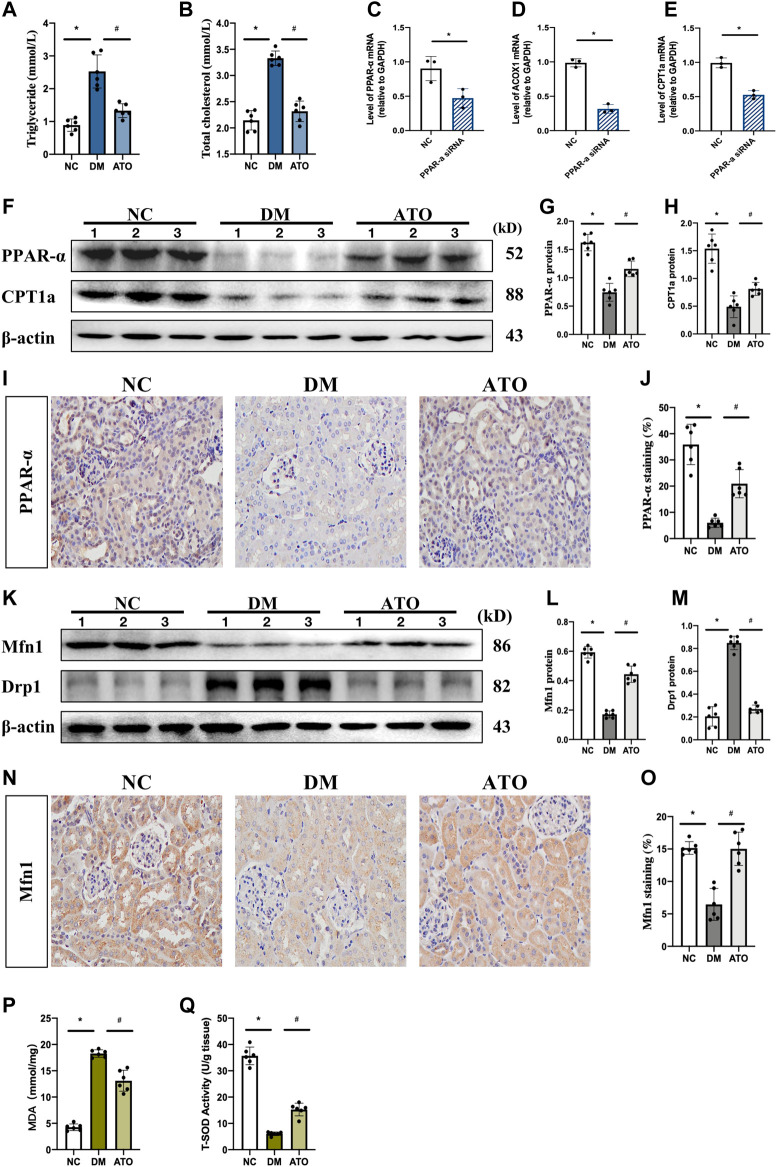
Atorvastatin restores PPARα expression and improves lipid metabolism and mitochondrial dysfunction in diabetic mice. Atorvastatin improves renal fibrosis and restores renal function in diabetic mice. The effects of Atorvastatin in blood. The experiments aimed to detect the changes in lipid metabolism and mitochondria-related indicators in each group. **(A, B)** Quantification of triglycerides **(A)** and total cholesterol **(B)** in three groups of kidney tissues. **(C–E)** Transfection of si-PPARα and its control sequence into mRTECs cells. qPCR was performed to detect the expression levels of PPARα **(C)**, and its downstream target genes ACOX1 **(D)** and CPT1a **(E)**. **(F–G)** Immunoblot analysis of PPARα, CPT1a in the three groups of kidney tissues, presenting quantitative data **(G, H)**. **(I–J)** Immunohistochemical staining **(I)**, and quantitative analysis **(J)** of PPARα. **(K–M)**. Immunoblotting for the detection of Mfn1 and Drp1 expression **(K)** and quantitative analysis of the results **(L, M)**. **(N–O)** Immunohistochemical staining **(N)**, and quantitative analysis **(O)** of Mfn1. **(P, Q)** Detection of malondialdehyde (MDA) content **(P)** and total superoxide dismutase (T-SOD) content **(Q)** in the three groups of kidney tissues. The *in vivo* experiments included the following groups: NC: normal diet-fed rats; DM: type 1 diabetic mice; ATO: type 1 diabetic mice treated with Atorvastatin. All data are indicative of mean ± SD from three independent experiments. n = 6; *p < 0.05 vs NC group. #*p* < 0.05, compared with the DM group.

The data indicated that plasma triglycerides and total cholesterol levels were significantly increased in DM mice, while the levels of these markers were significantly decreased in the ATO group. The kidney is a metabolically active tissue that uses fatty acids (FA) as a major energy source, PPARα, a key transcription factor of the fatty acid oxidation (FAO) pathway, can ameliorate the development of renal fibrosis (Wang, 2010; Su et al., 2020). The data indicated that the mRNA levels of carnitine palmitoyltransferase 1a (CPT1a) and acyl-coenzyme A oxidase 1 (ACOX1), which are genes related to lipid metabolism and play an important role in FA synthesis and TG accumulation, were significantly reduced following *in vitro* knockdown of PPARα expression (Figures 2C–E). *In vivo* experiments indicated that the levels of PPARα and CPT1a proteins were significantly reduced in the kidneys of DM mice. IHC further confirmed the decrease in PPARα levels, which was mainly expressed in the nucleus of renal tubular epithelial cells. Its expression was restored following administration of Atorvastatin (Figures 2I,J). These data combined with the results of the biochemical indices demonstrated that DM mice had developed lipid metabolism disorders, and that Atorvastatin could effectively improve these lipid metabolism disorders caused by insulin deficiency.

Mfn1 promotes mitochondrial fusion and maintains ATP production, while Drp1 mediates mitochondrial separation (Galvan et al., 2017). Both of the proteins are regulated by PPARα (Zolezzi et al., 2013). In the DM group, Mfn1 expression was decreased and Drp1 was increased. Following administration of Atorvastatin, the expression levels of Mfn1 were increased and those of Drp1 were decreased (Figures 2K–M). IHC results further indicated that Mfn1 expression in each group was mainly located in the cytoplasm of renal tubular epithelial cells (Figures 2N,O). These findings suggested that Atorvastatin could balance mitochondrial dynamics. The changes in mitochondrial dynamic can directly affect mitochondrial function, while ROS and total antioxidant capacity can reflect mitochondrial function to a certain extent. The levels of malondialdehyde (MDA) and total superoxide dismutase (T-SOD) represent the degree of lipid peroxidation and the antioxidant capacity of the cell under free radical attack, respectively. An increase in MDA levels and a decrease in antioxidant capacity was noted in the kidney tissues of DM mice (Figures 2P,Q), while MDA levels were decreased and the antioxidant capacity was increased in the ATO group. It was suggested that Atorvastatin could improve mitochondrial function and reduce the production of ROS and its peroxidative effects.

### PPARα is a Downstream Target Gene of miR-21, and Their Expression Levels are Negatively Correlated With the Progression of DKD

**FIGURE 3:**
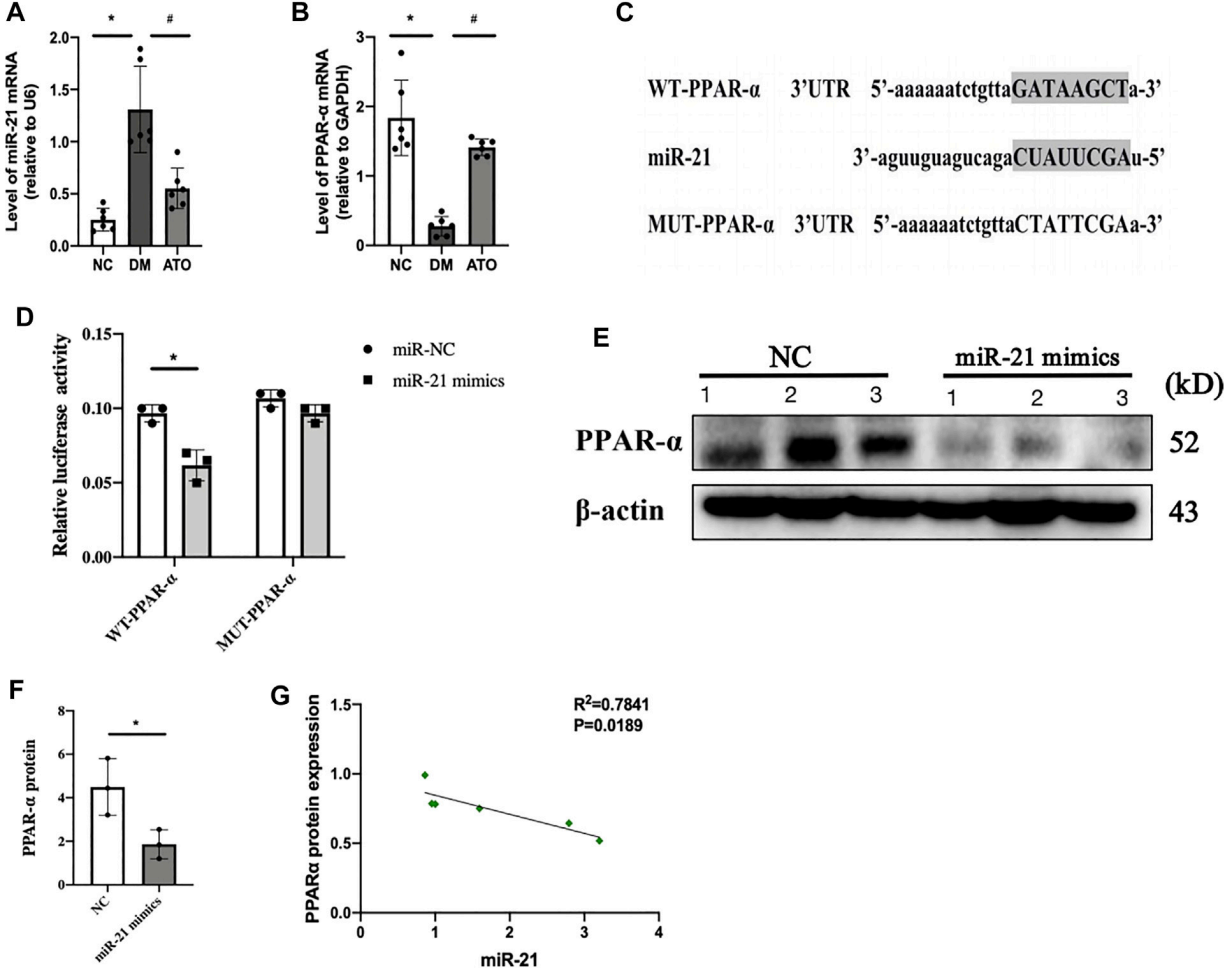
miR-21 directly targets and inhibits PPARα expression. The effects of Atorvastatin on specific blood parameters. The experiments aimed to detect the changes in lipid metabolism and mitochondria-related indicators in each group. miR-21 directly targets and inhibits PPARα expression. **(A, B)** The expression levels of miR-21 **(A)** and PPARα mRNA **(B)** were detected in the kidney tissues of mice in each group. n = 6; *p < 0.05 vs NC group. #*p* < 0.05, compared with the DM group. **(C)** Predicted binding sequences and mutation sites between miR-21 and PPARα 3′UTR seed sites. **(D)** Cotransfection of HK-2 cells with 50 nM miR-21 mimic or NC mimic and 100 ng of WT or MUT plasmid containing PPARα 3′UTR and 5 ng of Renilla plasmid. The dual-luciferase reporter assay was used to detect luciferase activity of WT and MUT plasmids. The data represent mean ± SEM, n = 3, *p < 0.05. (E. F) The expression levels of PPARα were detected by western blot analysis 24 h following transfection with miR-22 or NC mimics **(E)**. ImageJ optical density analysis of protein levels **(F)**. **(G)** Correlation analysis between miR-21 expression and PPARα protein expression. All data represent mean ± SD from three independent experiments. n = 3; *p < 0.05 vs NC group. #*p* < 0.05, compared with the DM group.

Bioinformatic analysis predicted that the seed sequence of miR-21 was complementary to the 3′ UTR sequence of PPARα mRNA (Figure 3C). In our previous study, it was shown that miR-21 expression was significantly increased in the DKD process and promoted fibrotic lesions in the renal tubular interstitium (Liu et al., 2019). In the present study, we detected a significant decrease in the protein (Figure 2F) and mRNA expression levels (Figure 3B) of PPARα. These changes were accompanied by a significant upregulation of miR-21 (Figure 3A) in the kidney tissues of DM mice, while Atorvastatin intervention significantly upregulated the protein and mRNA expression of PPARα and downregulated its miR-21 expression. The experiments further verified that PPARα was the target gene of miR-21 by the dual-luciferase reporter assay, and the results indicated that miR-21 could regulate PPARα mRNA levels. When the cells were transfected with wild-type vector WT-PPARα in HK-2 cells, the luciferase activity in the miR-21 group was significantly lower than that in the miR-NC group. However, when the cells were transfected with mutant vector MUT-PPARα, the luciferase activity in the miR-21 group was not statistically significant compared with that of the miR-NC group (Figure 3D). In addition, transfection of miR-21 mimics into HK-2 cells resulted in a significant decrease in PPARα protein levels (Figures 3E,F). Correlation analysis indicated that miR-21 expression demonstrated a negative correlation with PPARα protein expression (Figure 3G). This is consistent with the findings noted in human liver and diabetic eye disease mice [32], indicating that PPARα is a target gene of miR-21.

### The miR-21 Inhibitor and Fenofibrate, an Exogenous Ligand of PPARα, Inhibit High-Glucose, High-Fat, and High-Glucose High-Fat-Mediated Renal Tubular Epithelial Cell Injury

**FIGURE 4:**
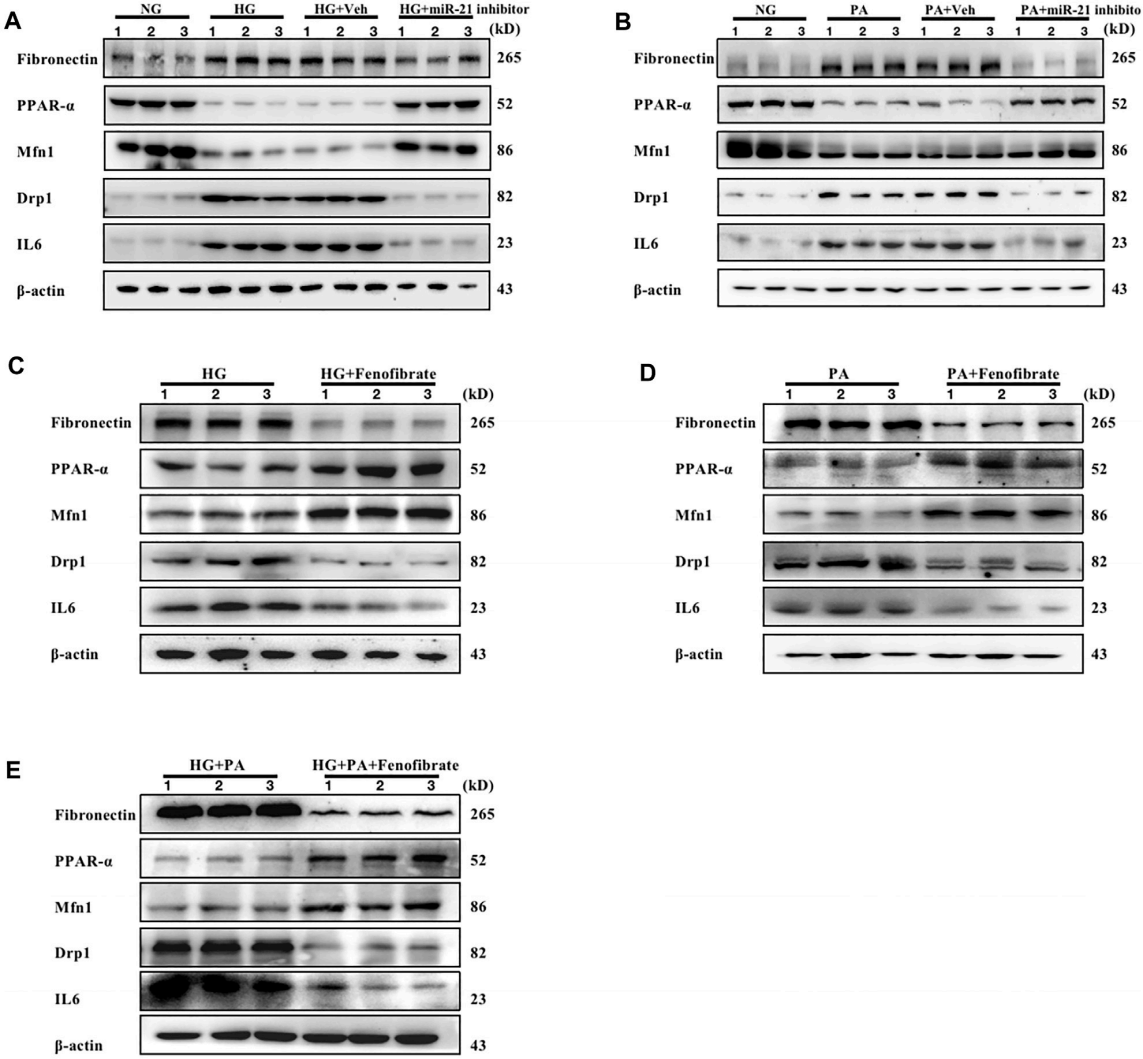
Both miR-21 inhibitors and Fenofibrate slow down renal tubular injury caused by high glucose or high fat. mRTEC cells were incubated with NG (5.5 mM) and HG (25 mM) medium, respectively, and transfected with miR-21 inhibitor and its control agent as (HG + Veh group, HG + miR-21 inhibitor group) in HG-treated cells for 48 h. **(A)** Immunoblotting bands corresponding to FN, PPARα, Mfn1, Drp1, and IL-6 expressions in RTEC cells of each group. mRTEC cells were treated with palmitic acid solvent. 95% anhydrous ethanol (0.02 ml) was added in the NC group and palmitic acid PA (0.2 mM) in the PA group (PA-treated). RTEC cells were transfected with miR-21 inhibitor and its control agent (PA + Veh group, PA + miR-21 inhibitor group) following 48 h of incubation. Subsequently, the samples were analyzed. **(B)** Immunoblotting bands corresponding to FN, PPARα, Mfn1, Drp1, and IL-6 expressions in RTEC cells of each group. **(C–E)** The expression levels of FN, PPARα, Mfn1, Drp1, and IL-6 in RTEC cells treated with HG, PA, and HG + PA, respectively, and subsequently incubated with Fenofibrate (0.05 mM) for 48 h. The expression levels of FN, PPARα, Mfn1, Drp1, and IL-6 proteins were analyzed under HG (F), PA (G), and HG + PA(H) conditions, respectively. All data are indicative of mean ± SD from three independent experiments. n = 3.

mRTEC cells were cultured with high glucose (HG) medium to assess the effects of the DM-induced high glucose environment on renal tubular cells. RTEC cells were cultured with high concentrations of palmitate (PA) to investigate the effects of DM-induced high fat environment on renal tubular cells. The cells were treated with transfected miR-21 inhibitor and the PPARα-specific agonist Fenofibrate, respectively. The results indicated that the protein levels of FN, IL-6, and Drp1 were increased in the cells cultured under HG and PA conditions, while the protein levels of PPARα and Mfn1 were decreased compared with those of the control group. However, following inhibition of miR-21 expression, the expression levels of FN, IL-6, and Drp1 proteins were decreased in cells cultured in the presence of either HG or PA, while the expression levels of PPARα and Mfn1 were increased (Figures 4A– B). Fenofibrate, which is a ligand of PPARα, is often used clinically as a PPARα-specific agonist. This compound exhibited similar effects to those of miR-21 inhibitors under different conditions of HG, PA, and HG + PA (Figures 4C–E). These findings suggest that both high-glucose or high-fat environments cause downregulation of PPARα expression, impair mitochondrial function, and promote the expression of inflammatory factors and fibronectin to mediate renal tubular injury. In contrast to these findings, miR-21 inhibitor and Fenofibrate both upregulated PPARα expression, which in turn restored the expression levels of proteins affecting mitochondrial function and reversed the inflammatory response and fibrosis progression in renal tubular epithelial cells induced by hyperglycemia and hyperlipidemia. These results suggested that miR-21 may contribute to high glucose or high fat-induced renal tubular epithelial cell injury by downregulating PPARα expression leading to impaired mitochondrial function and lipid metabolism disorders.

### Atorvastatin Restores PPARα Expression by Inhibiting miR-21 Expression and Improves Mitochondrial Function Impairment in Renal Tubular Epithelial Cells Induced by High Glucose and High Fat

**FIGURE 5:**
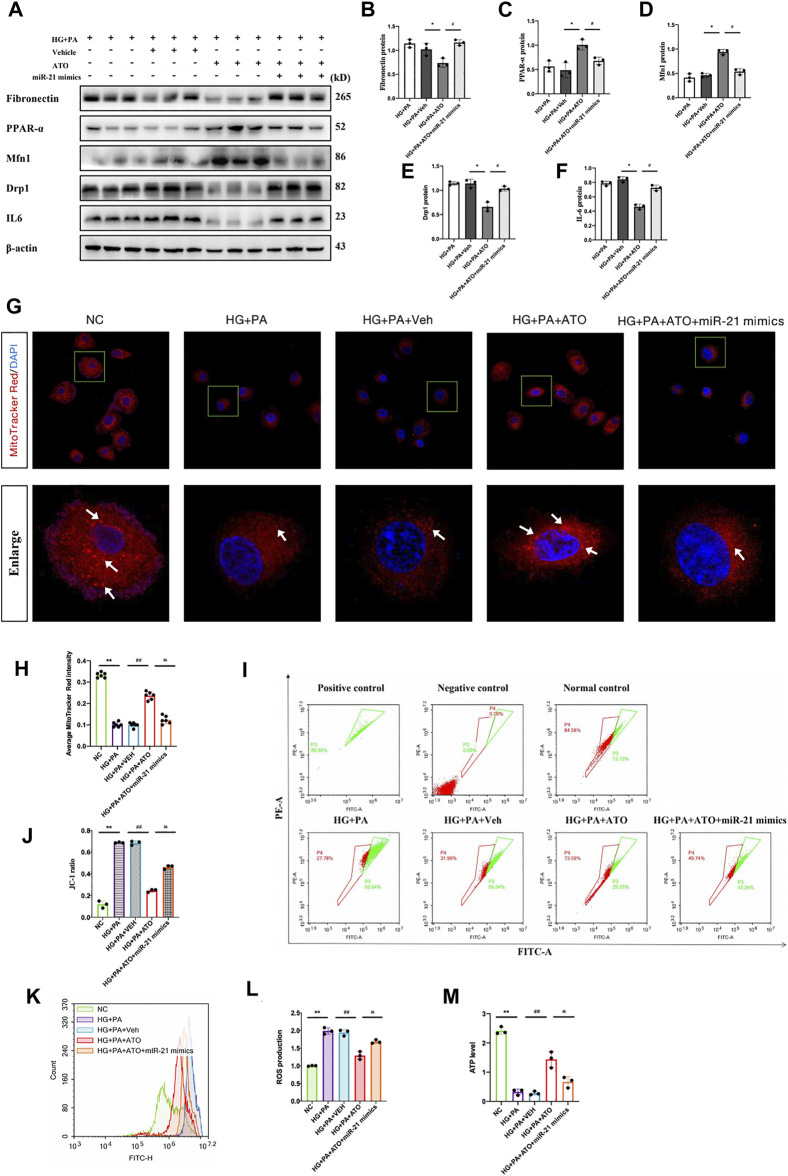
Atorvastatin restores PPARα expression by inhibiting miR-21 expression and improves mitochondrial function impairment in renal tubular epithelial cells induced by high glucose and high fat. The mRTEC cells were cultured with NG and HG + PA medium, and Atorvastatin and its solvent DMSO were added to the HG + PA-treated cells as the treatment group and treatment control group, respectively. The cells were also transfected with miR-21 mimics and treated with HG + PA + ATO. (A-F)They were subsequently cultured for 48 h. Immunoblot bands **(A)** and quantitative data **(B–F)** of FN, PPARα, Mfn1, Drp1, and IL-6 in each cell group. **(G)** Representative confocal microscope images of mitochondrial morphology stained by MitoTracker Red. Original magnification ×600. **(H)** Quantification of mitochondrial number per cell. White arrows represent high fluorescence intensity. **(I–J)** Flow cytometry analysis of mitochondrial membrane potential changes in each group of cells **(I)**, quantitative data **(J)**. **(K–L)** Flow cytometry analysis of ROS production **(K)**, quantitative data **(L)** for each group of cells. **(M)** Assessment of the ATP content in the cells of each group. All data are mean ± SD from three independent experiments. n = 3; *p < 0.05 vs NC group. #*p* < 0.05, compared with the HG + PA group. *p* < 0.05, compared with the HG + PA + ATO group; **p < 0.01 vs NC group. ##*p* < 0.01, compared with the HG + PA group. *p* < 0.01, compared with the HG + PA + ATO group.

Subsequent experiments were performed to clarify whether Atorvastatin restores the transcription and protein expression of PPARα, which is a key factor in lipid metabolism. It was hypothesized that Atorvastatin could downregulate miR-21 expression, thereby improving mitochondrial function and inhibiting the damage to renal tubular epithelial cells caused by high glucose and high fat. In order to confirm this hypothesis, we transfected miR-21 mimics into RTEC cells following treatment of the cells with high glucose and high fat in the presence of Atorvastatin. The expression levels of PPARα were evaluated, in combination with mitochondrial kinetics and function, and the induction of the inflammatory response was assessed. The high glucose and high-fat environment led to a decrease in PPARα levels, a decrease in the protein expression levels of Mfn1, which is an important protein in mitochondrial dynamics, and an increase in Drp1 levels, which were accompanied by fibrotic damage and inflammatory response (Figure 5A); it also led to reduced the number of biologically active mitochondria and ATP content, mitochondrial depolarization, and a significant increase in ROS production (Figures 5H–M). In contrast to these observations, Atorvastatin caused an upregulation in the protein expression levels of PPARα and Mfn1, a reduction in the expression levels of Drp1, FN, and IL6, and in the levels of ROS and led to restoration of the ATP content, mitochondrial activity, and polarization response. Following transfection of the cells with miR-21 mimics and treatment of Atorvastatin, the aforementioned effects of this compound were significantly inhibited. This suggested that miR-21 significantly inhibited the restoration of PPARα expression by Atorvastatin, improved HG- and PA-induced mitochondrial dysfunction, and reversed the inflammatory response and fibrosis progression effects in renal tubular epithelial cells cultured under hyperglycemic and hyperlipidemic conditions. Therefore, it is suggested that Atorvastatin exhibits a protective effect on mitochondrial dysfunction in renal tubular epithelial cells treated with high glucose and lipids by inhibiting miR-21 expression and restoring PPARα expression.

## Discussion

The kidneys require large amounts of mitochondria to remove waste products from the blood and to regulate fluid and electrolyte balance. Following mitochondrial dysfunction, a reduction in the ATP production is noted, in combination with altered cell function and structure, and loss of kidney function. These changes eventually lead to the progression of the chronic kidney disease (CKD). Restoring mitochondrial function may subsequently reverse cellular damage and restore renal function. In recent years, studies on mitochondrial dysfunction in diabetic nephropathy have focused on hyperglycemia-induced ATP depletion, which triggers changes in mitochondrial morphology. However, type 1 diabetes mellitus (T1DM) can also lead to dyslipidemia and proteinuria (Vergès, 2020). Therefore, in type 1 diabetes, the disorders in glucose metabolism caused by islet damage are the only factors responsible for the progression of the disease. Specific conditions, such as alterations in lipid transport, lipid storage, and membrane lipids can affect mitochondrial function (Ducasa et al., 2019). For example, the accumulation of FA in acute kidney injury and diabetic nephropathy leads to decreased β-oxidation in mitochondria and increased formation of intracellular lipid droplets, which inhibit ATP production (Bhargava and Schnellmann, 2017)and exacerbate kidney damage. Therefore, improving mitochondrial function plays a major role in combating acute and chronic injury.

Statins, also known as hydroxymethylglutaryl coenzyme A (HMG-CoA) reductase inhibitors, demonstrate a competitive mechanism to inhibit endogenous HMG-CoA reductase, lowering cholesterol and triglyceride and exerting a lipid-regulating effect (Xu and Wu, 2020). Atorvastatin, is a highly effective statin lipid-lowering drug that has been widely used in clinical practice. The current research on Atorvastatin is mainly focused on its cardiovascular protective effects, in addition to its significant lipid-lowering efficacy. It has been reported that statins can exert renoprotective effects, such as anti-inflammatory and antifibrotic effects through the activation of various pathways (Pose et al., 2019). However, the renoprotective mechanism of Atorvastatin in diabetic nephropathy, which is caused via hypolipidemic effects has not been previously clarified. In the present study, the data indicated that T1DM mice exhibited significant disorders in glucolipid metabolism, whereas their kidney morphology exhibited apparent pathological changes, such as widening of the thylakoid zone, collagen deposition, and lymphocyte infiltration. The expression levels of the fibrosis-related proteins fenonectin, collagen-I, a-SMA, and inflammatory factor IL-6 were significantly increased in the kidney tissues of DM mice. The triglyceride and total cholesterol levels were reduced in the ATO group of mice following administration of Atorvastatin in DM mice. Although the blood glucose levels of DM mice did not change significantly following drug administration, the expression levels of fibrotic proteins and inflammatory factors were significantly reduced. Moreover, the renal function was restored. Their pathological morphology was significantly improved and the area of collagen deposition was reduced. Our results clearly indicated that Atorvastatin was effective in slowing down the progression of T1DM mice, which prompted us to investigate the mechanism by which inhibition of lipid metabolism disorder can alleviate the progression of DKD.

Recently, the role of the impaired fatty acid oxidation (FAO) pathway in the development of renal interstitial fibrosis has been reported in the literature (Kang et al., 2015). It has been shown that renal tubular epithelial cells were heavily dependent on FAO, which was their main energy source. Moreover, diminished FAO was associated with intracellular lipid accumulation. PPARα is a key transcription factor of FAO and it is involved in oxidative metabolism noted in the majority of the tissues, since it activates numerous genes involved in the following pathways: Carnitine palmitoyltransferase 1 a (CPT1a), acyl-coenzyme A oxidase 1 (ACOX1), acyl-coenzyme A dehydrogenase (ADH), and mitochondrial thioesterase 1 (MTE1) (Grabacka et al., 2013). Our results also validated that downregulation of PPARα expression suppressed the expression levels of CPT1a with ACOX1. Increasing evidence supports a link between PPARα and the incidence of metabolic diseases including diabetes, obesity, dyslipidemia and fatty liver. Therefore, the role of PPARα in the development of renal diseases has recently been extensively studied (Wang, 2010), such as in mouse kidney tissues with hyperlipidemia (Chung et al., 2018)and renal stone disease (Su et al., 2020). Low expression of PPARα leads to lipid accumulation in renal tubular cells, which causes lipotoxicity and can manifest as mitochondrial dysfunction associated with increased reactive oxygen species production and decreased ATP production, apoptosis, and elevated inflammatory cytokine dysfunction (Jao et al., 2019). Therefore, we examined the changes in PPARα transcription and protein expression in mouse kidney tissues. In addition, we examined the role of the FAO-rate-limiting enzyme CPT1, whose protein levels were downregulated in DM mice compared with those of the control mice. These changes also reduced PPARα transcripts and protein expression levels. Following administration of Atorvastatin, PPARα and CPT1 expression were significantly increased. Recent studies have highlighted the importance of mitochondrial fusion and fission in cell function and animal physiology (Detmer and Chan, 2007). For example, fibroblasts lacking Mfn1 completely did not indicate mitochondrial fusion and exhibited severe cellular defects, including poor growth and heterogeneity of mitochondrial membrane potential (Chen et al., 2005). In contrast to these findings, downregulation of Drp1 expression reversed palmitate-induced mitochondrial damage and ROS production in skeletal muscle cells (Jheng et al., 2012). It has also been reported that the PPARα agonist (WY 14.643) was able to protect neurons by regulating mitochondrial fusion and fission in the brain of a transgenic mouse model of Alzheimer’s disease (Zolezzi et al., 2013). Therefore, balanced mitochondrial dynamics are essential to maintain mitochondrial function and energy production. In the present study, we examined the expression levels of these two important proteins, Mfn1 and Drp1, which directly affected mitochondrial function. The expression levels of Mfn1 were decreased and those of Drp1 were increased in DM mice, whereas the expression levels of both of these proteins were reversed following administration of Atorvastatin. This implies that Atorvastatin exhibits antilipidemic effects that directly or indirectly balance mitochondrial dynamics and protect its function, in addition to its regulatory effects on lipid metabolism disorders.

MicroRNAs (miRNAs) are single-stranded, non-coding small RNA molecules that negatively regulate gene expression by binding to the 3′ untranslated region (UTR) of the target mRNA. This in turn regulates a variety of biological and pathological processes (Filipowicz et al., 2008). The results of the bioinformatics analysis suggested that miR-21 exhibited specific binding sites for PPARα. We observed elevated miR-21 expression and decreased PPARα expression in T1DM mice. Increased miR-21 expression significantly decreased PPARα protein levels. The luciferase reporter gene assay confirmed that PPARα was a downstream target gene of miR-21, which was consistent with the findings of Su B in a mouse model of renal stones with renal calcium oxalate deposition (Su et al., 2020). To simulate the damaging effects of high glucose and high-fat environments on renal tubular epithelial cells *in vivo*, renal tubular epithelial cells were treated with high glucose and palmitate, a saturated fatty acid commonly used as a diabetic stressor. The results indicated induction of oxidative stress leading to cellular dysfunction and apoptosis in various cell types (Cacicedo et al., 2005; Staiger et al., 2006). Moreover, it was shown that high sugar and palmitate levels contributed to decreased PPARα expression, whereas inhibition of miR-21 expression increased PPARα expression and protected mitochondrial dynamics leading to reduction of fibrosis and the inflammatory response.

Fenofibrate is a specific agonist of PPARα and is used clinically to reduce lipid levels in patients with dyslipidemia and cardiovascular disease (McKeage et al., 2011). Fenofibrate was administered to mRTECs subjected to high glucose, high fat, and high glucose combined with high fat conditions, respectively. The data indicated similar effects with those of the miR-21 inhibitors. These results described for the first time the relationship between the PPARα agonist Fenofibrate and mitochondrial dynamics and confirmed that by regulating PPARα expression, which is the key factor of lipid metabolism, mitochondrial dynamics and the corresponding protein expressions are affected. Concomitantly, the inflammatory response and fibrous deposition were inhibited, exerting a protective effect against DKD.

To further explore the association and role of Atorvastatin, the expression levels of miR-21, PPARα, and the mitochondrial function were assessed in DKD. Following administration of Atorvastatin in RTECS treated under high fat and high glucose conditions, the cells were transfected with miR-21 mimics. We found that administration of Atorvastatin significantly reduced the effects of high glucose conditions, whereas the combination with high fat conditions increased PPARα expression and altered the expression levels of two important proteins involved in mitochondrial dynamics. Specifically, Mfn1 levels were increased and Drp1 levels were decreased. Following transfection of the cells with miR-21 mimics and Atorvastatin administration, the protective effect of this drug was significantly inhibited and mitochondrial function was again impaired. This further confirmed our previous hypothesis that Atorvastatin improves mitochondrial function by downregulating miR-21-mediated-inhibition of lipid metabolism disorders to alleviate the progression of diabetic nephropathy.

In conclusion, Atorvastatin plays a protective role in the pathogenesis of DKD by promoting the expression of PPARα, a key transcription factor regulating lipid metabolism. In addition, it improved mitochondrial function and inflammatory response to counteract renal tubulointerstitial fibrosis. The specific mechanism involved the ability of Atorvastatin to promote PPARα transcription and expression by downregulation miR-21 expression, while restoring mitochondrial function to maintain the structure and function of renal tubular epithelial cells.

## Data Availability

The original contributions presented in the study are included in the article/Supplementary Materials, further inquiries can be directed to the corresponding authors.

## Abbreviations

DKD, Diabetic kidney disease; RTECs, Renal tubular epithelial cells; PPARα, Peroxisome proliferator-activated receptors-α; FN, Fibronectin; Mfn1, Mitofusin1; Drp1, dynamin-like protein 1; IL-6, Interleukin-6; STZ, Streptozocin; FA, Fatty acids; T1D, Type 1 diabetes; PAS, Periodic acid-Schiff; DMEM, Dulbecco’s modified Eagle’s medium; FBS, Fetal bovine serum; NG, Normal glucose; HG, High glucose; PA, Palmitic acid; α-SMA, α-smooth muscle actin; Col III, Collagen III.
