# Supplementary figures and images for: Atorvastatin Restores PPARα Inhibition of Lipid Metabolism Disorders by Downregulating miR-21 Expression to Improve Mitochondrial Function and Alleviate Diabetic Nephropathy Progression
Source: Front Pharmacol. 2022 Feb 11;13:819787. doi: 10.3389/fphar.2022.819787 (PMC8874267; doi:10.3389/fphar.2022.819787)

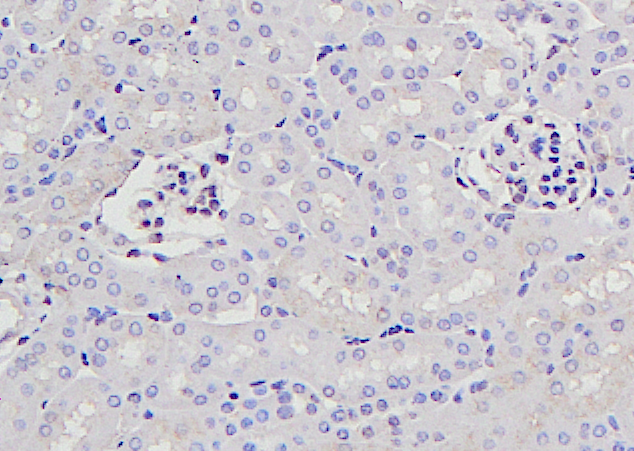

Supplement: Supplementary file 1 [file DataSheet3.ZIP › the original Images,fig2/IHC PPARa/DM.tif]

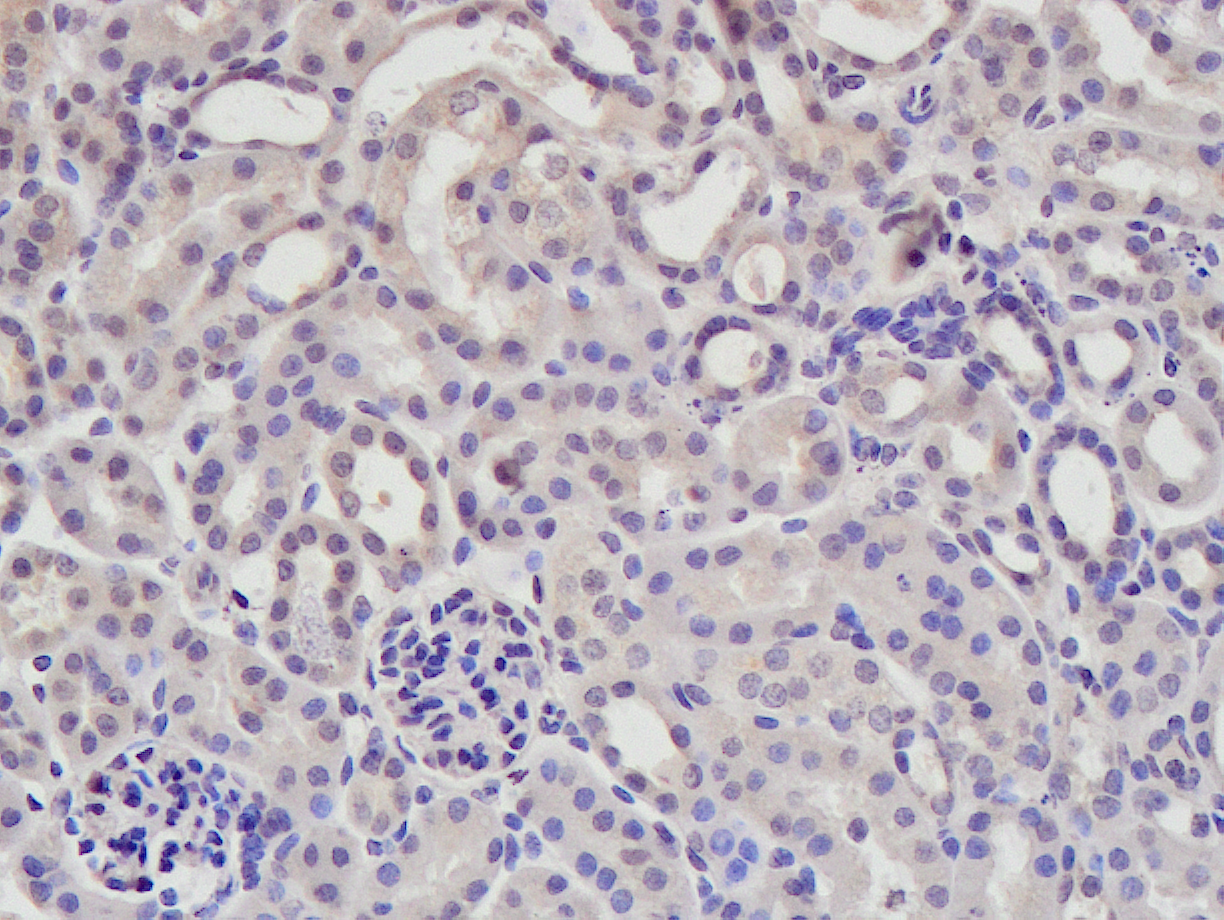

Supplement: Supplementary file 1 [file DataSheet3.ZIP › the original Images,fig2/IHC PPARa/ATO.tif]

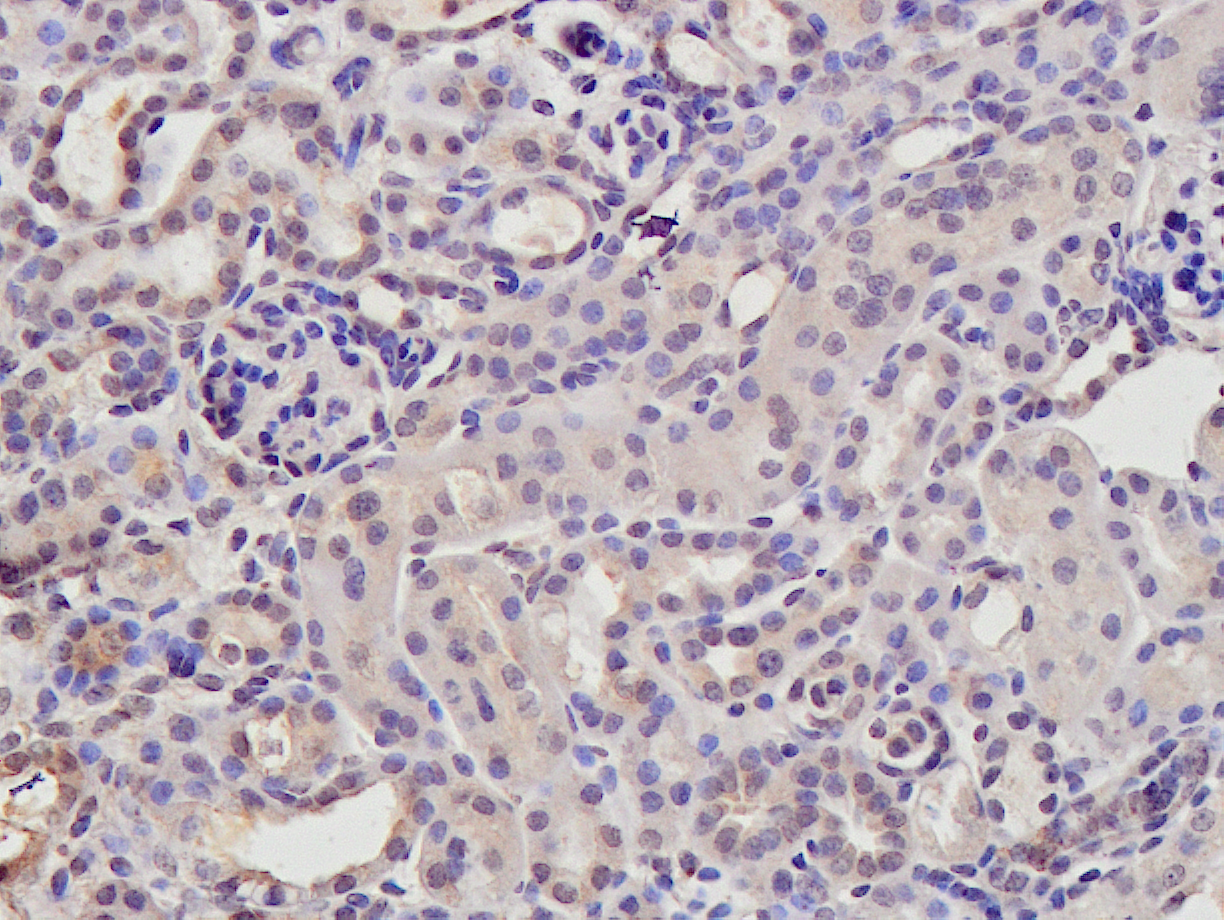

Supplement: Supplementary file 1 [file DataSheet3.ZIP › the original Images,fig2/IHC PPARa/NC.tif]

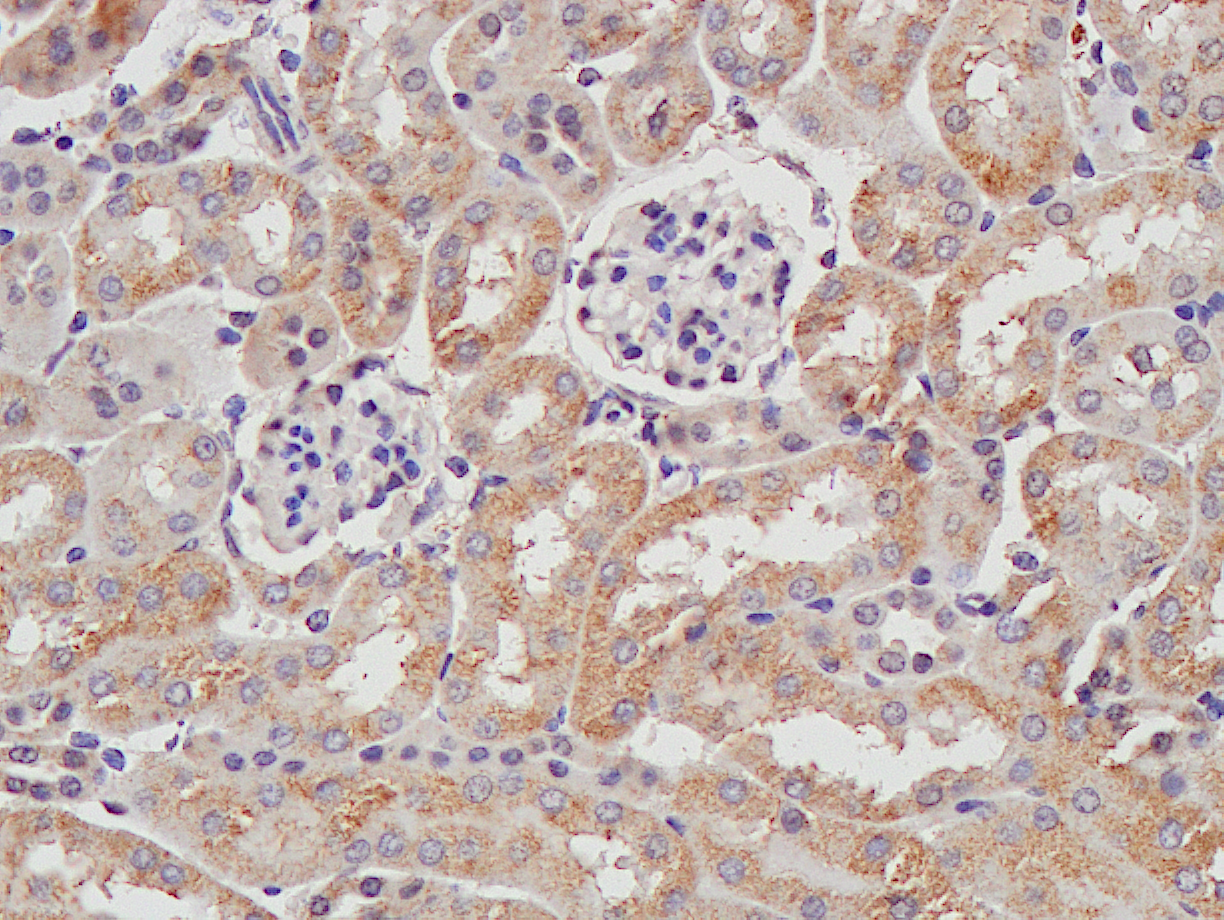

Supplement: Supplementary file 1 [file DataSheet3.ZIP › the original Images,fig2/IHC Mfn1/NC 1 400.tif]

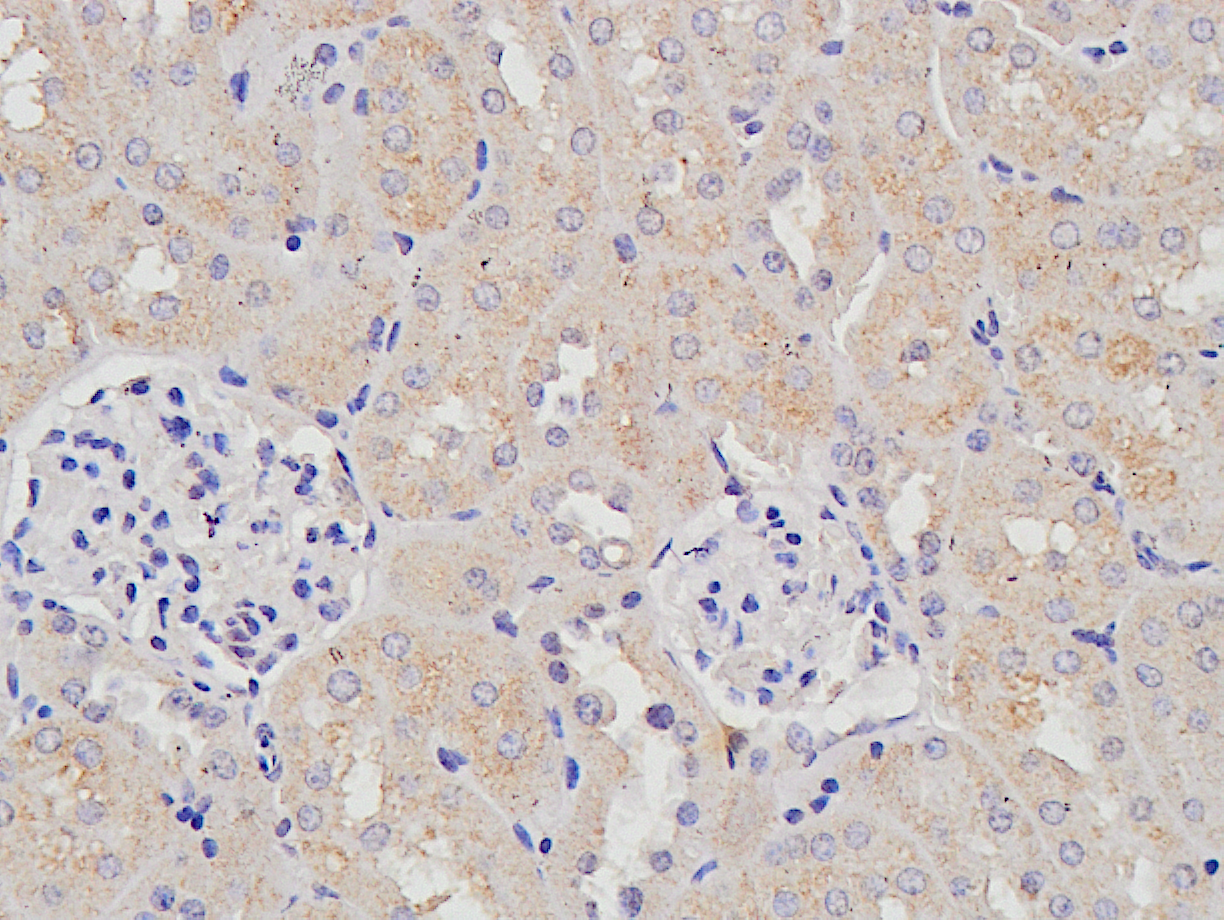

Supplement: Supplementary file 1 [file DataSheet3.ZIP › the original Images,fig2/IHC Mfn1/DM1 400.tif]

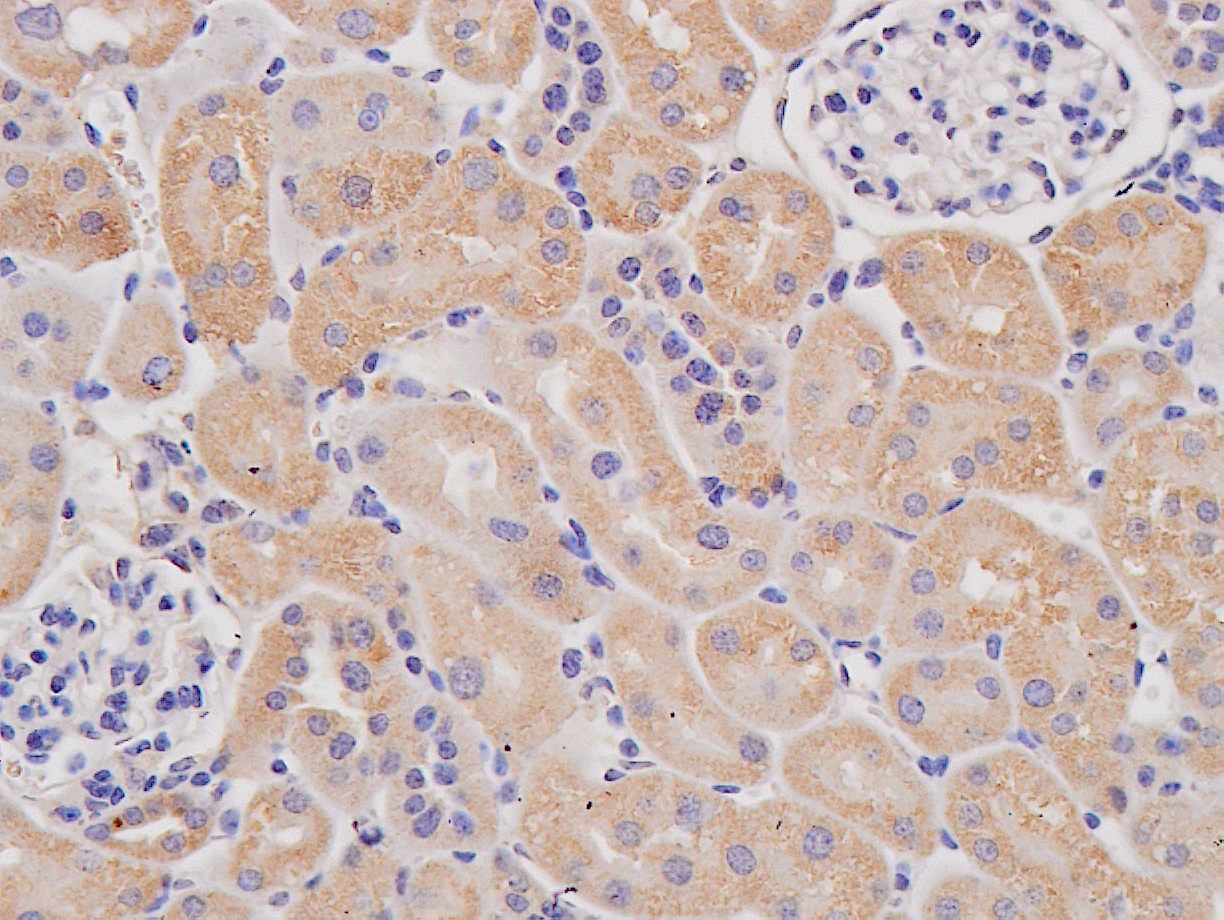

Supplement: Supplementary file 1 [file DataSheet3.ZIP › the original Images,fig2/IHC Mfn1/ATO1 400.tif]

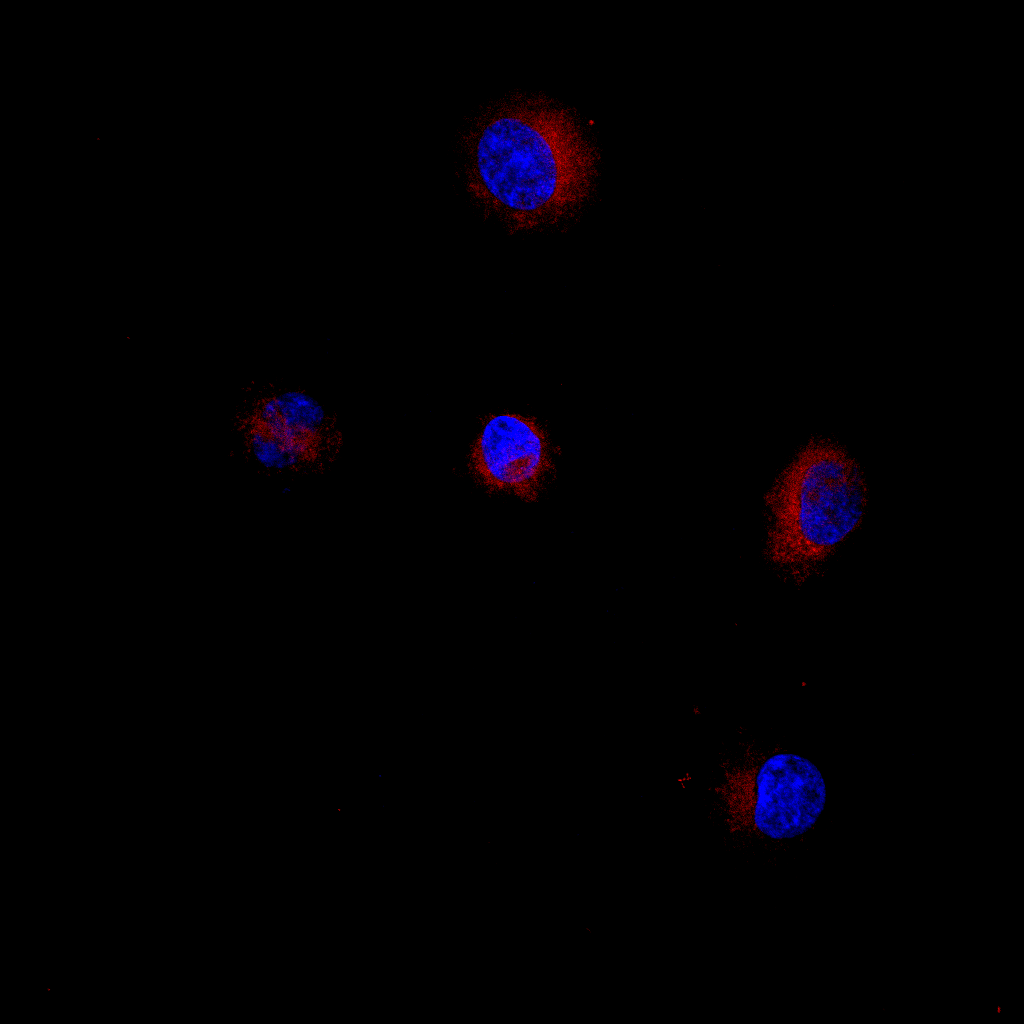

Supplement: Supplementary file 2 [file DataSheet4.ZIP › Supplementary experimental original data/images/HG+PA+ATO+miR-21 mimics.tif]

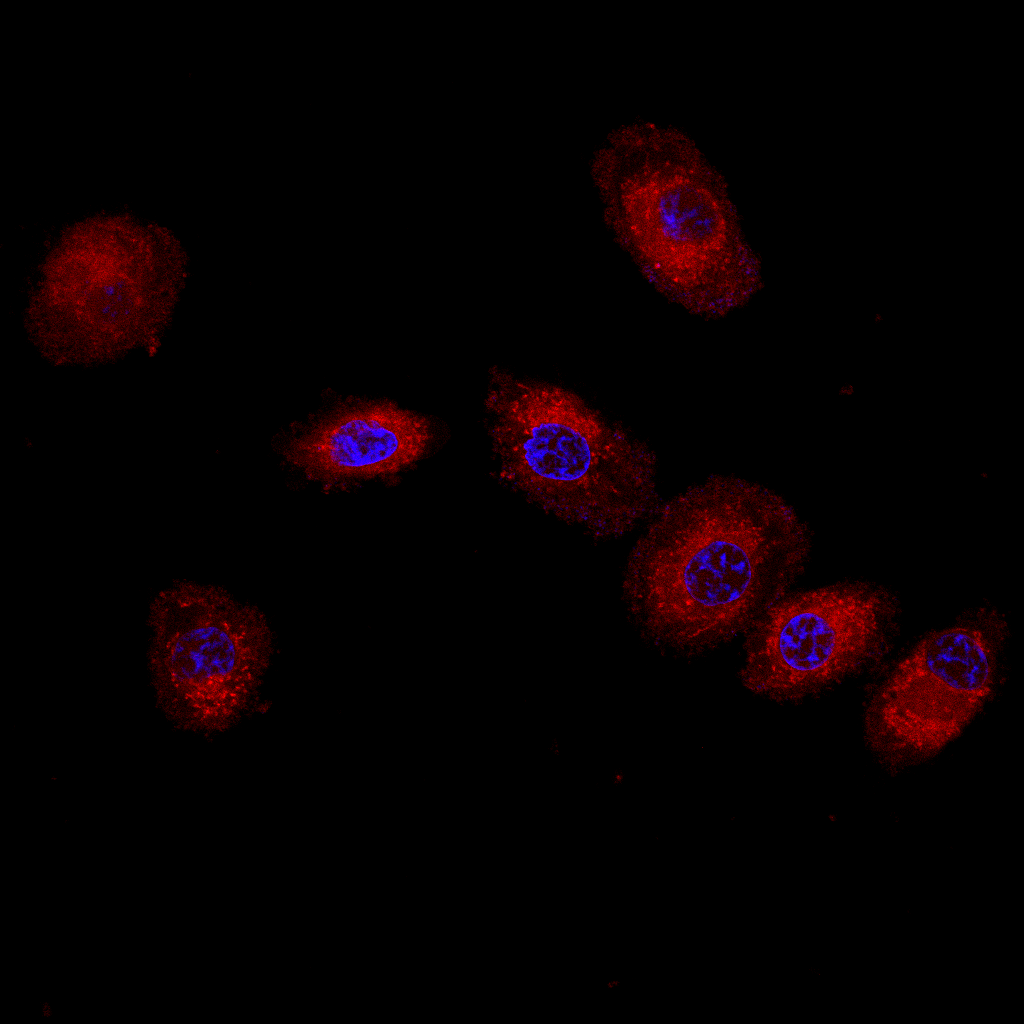

Supplement: Supplementary file 2 [file DataSheet4.ZIP › Supplementary experimental original data/images/HG+PA+ATO.tif]

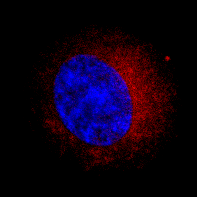

Supplement: Supplementary file 2 [file DataSheet4.ZIP › Supplementary experimental original data/images/enlarge/HG+PA+ATO+miR-21 mimics.tif]

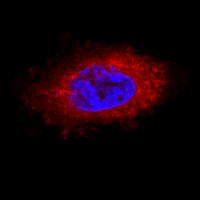

Supplement: Supplementary file 2 [file DataSheet4.ZIP › Supplementary experimental original data/images/enlarge/HG+PA+ATO.tif]

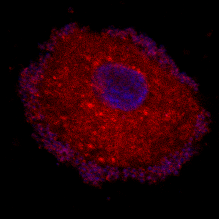

Supplement: Supplementary file 2 [file DataSheet4.ZIP › Supplementary experimental original data/images/enlarge/NC.tif]

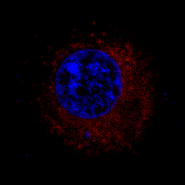

Supplement: Supplementary file 2 [file DataSheet4.ZIP › Supplementary experimental original data/images/enlarge/HG+PA+Veh.tif]

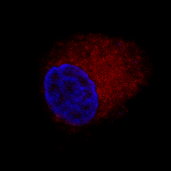

Supplement: Supplementary file 2 [file DataSheet4.ZIP › Supplementary experimental original data/images/enlarge/HG+PA.tif]

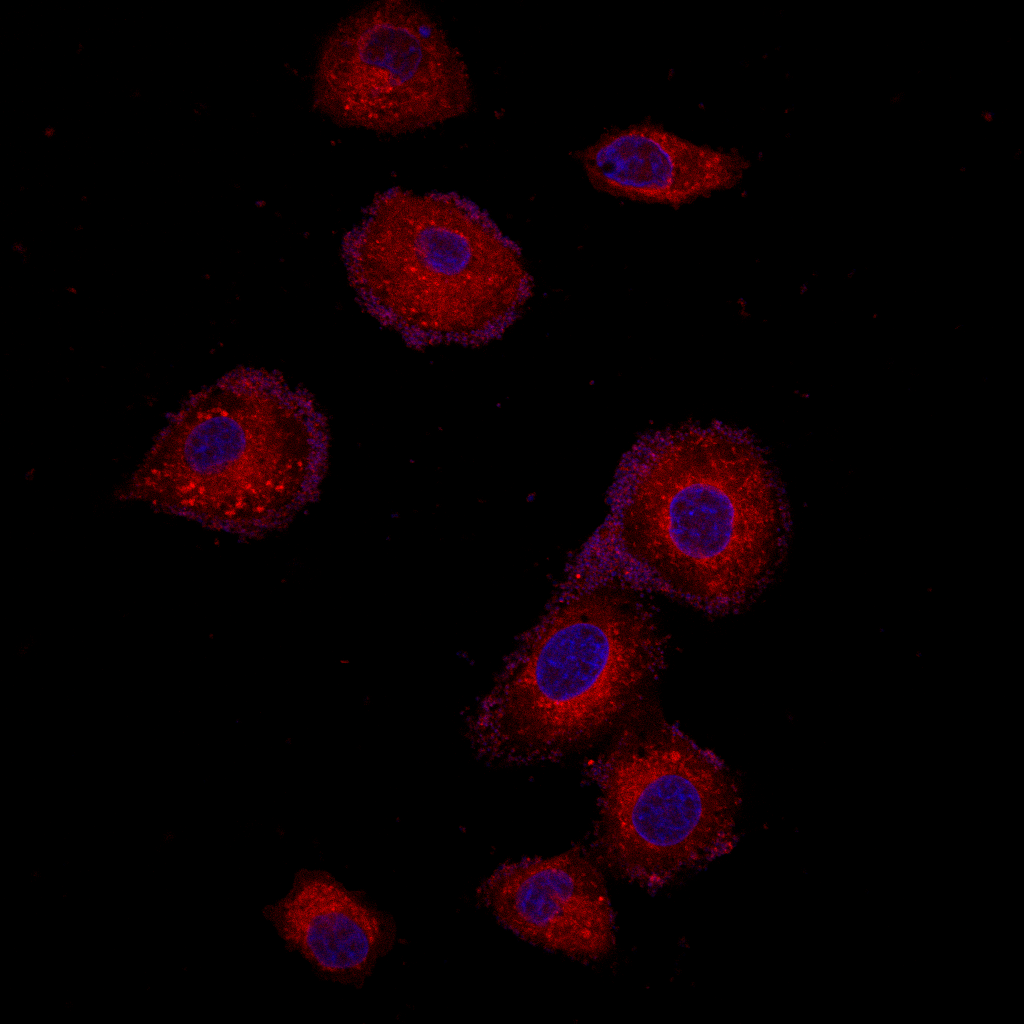

Supplement: Supplementary file 2 [file DataSheet4.ZIP › Supplementary experimental original data/images/NC.tif]

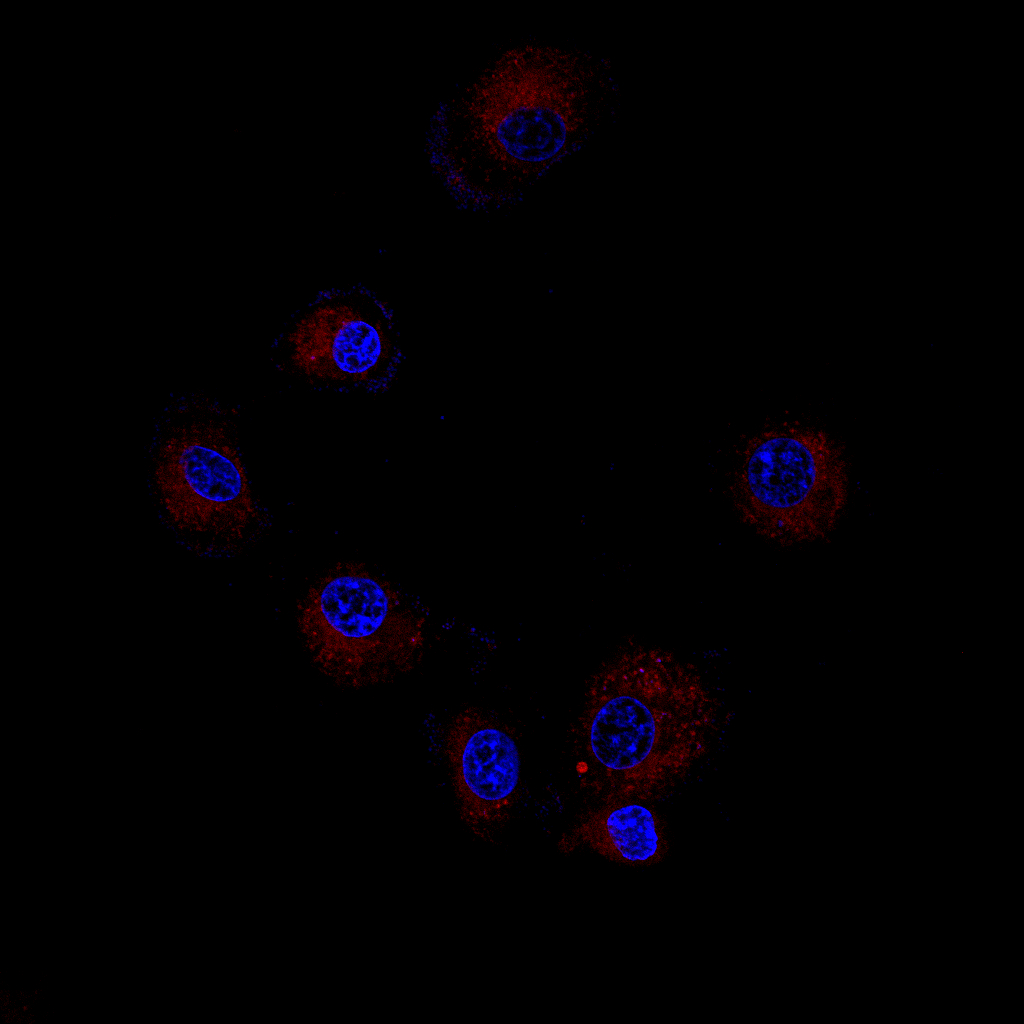

Supplement: Supplementary file 2 [file DataSheet4.ZIP › Supplementary experimental original data/images/HG+PA+Veh.tif]

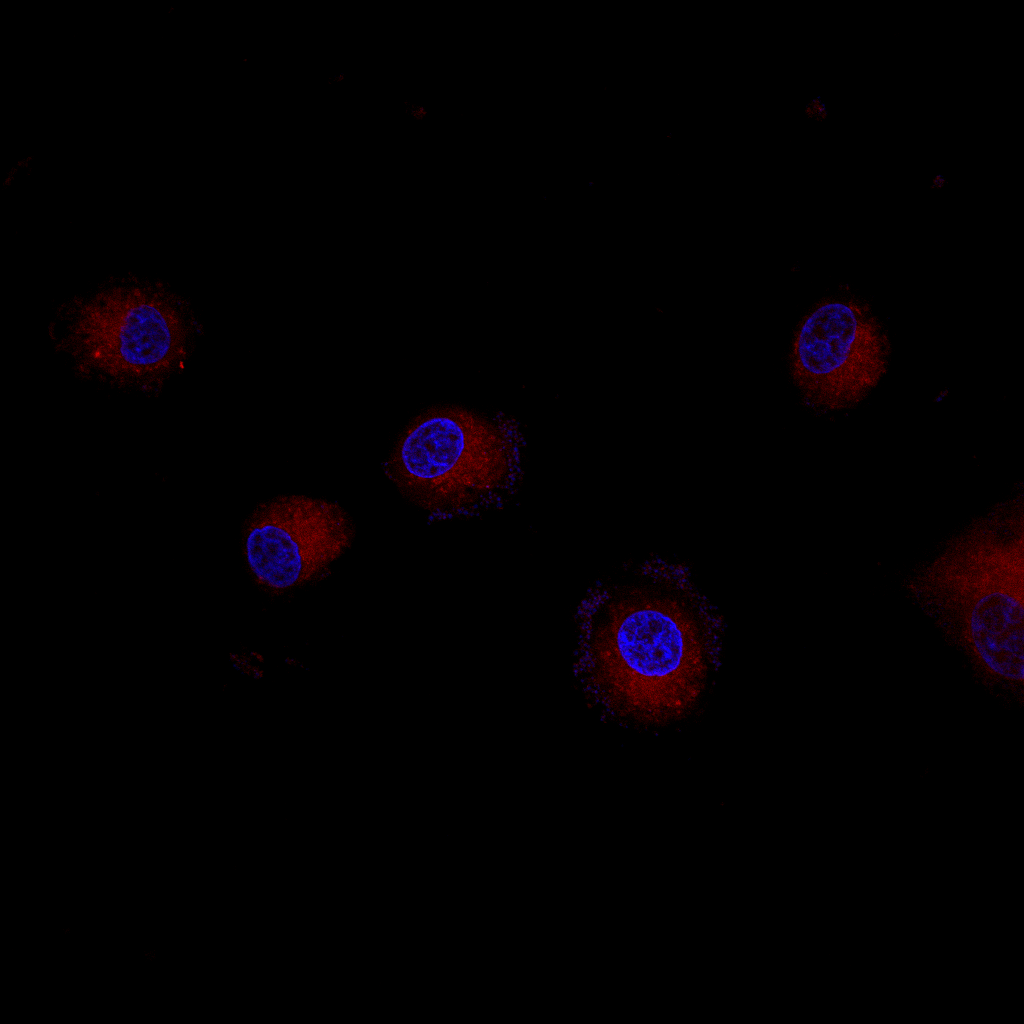

Supplement: Supplementary file 2 [file DataSheet4.ZIP › Supplementary experimental original data/images/HG+PA.tif]

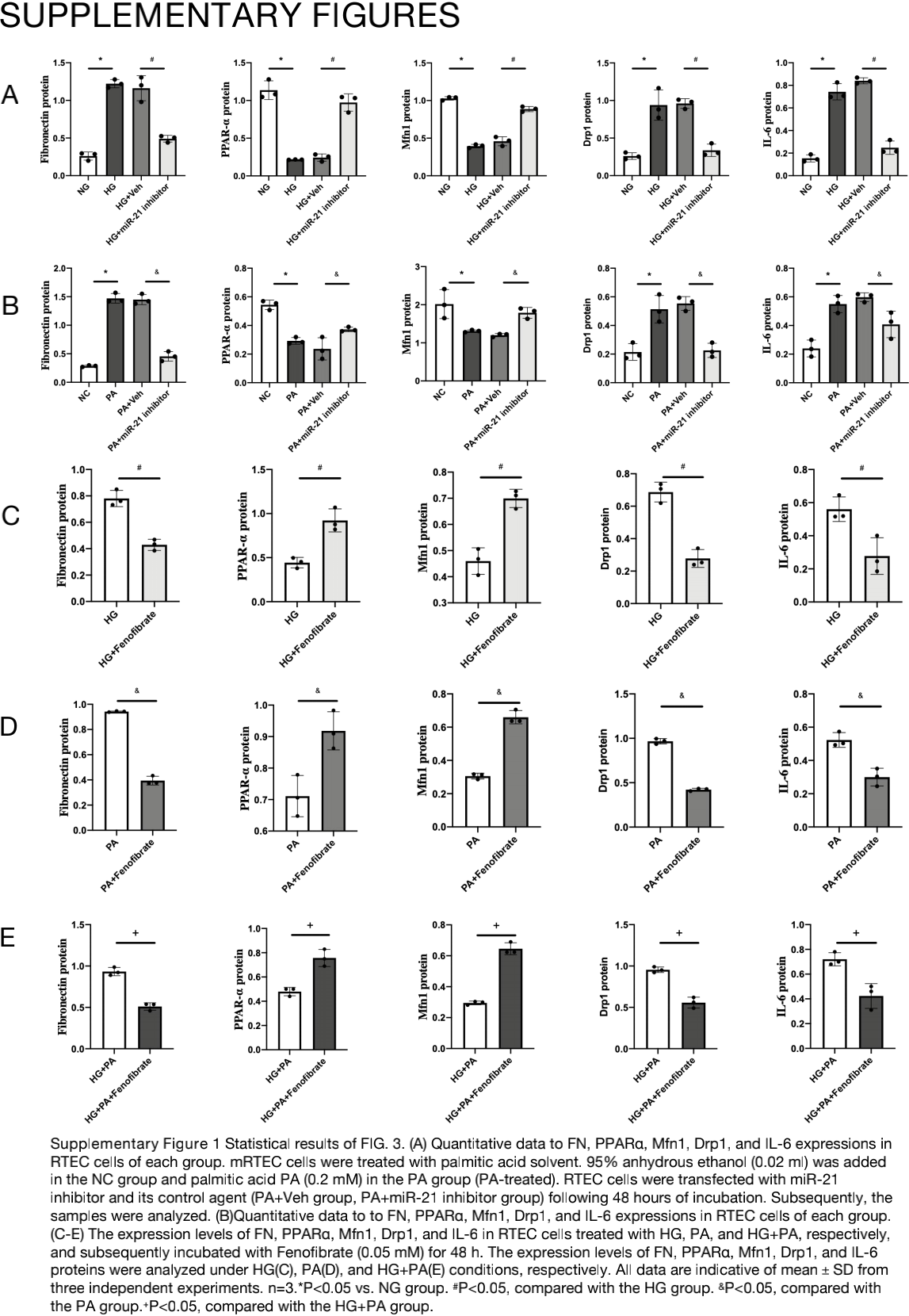

Supplement: Supplementary file 4 [file Image1.TIF]

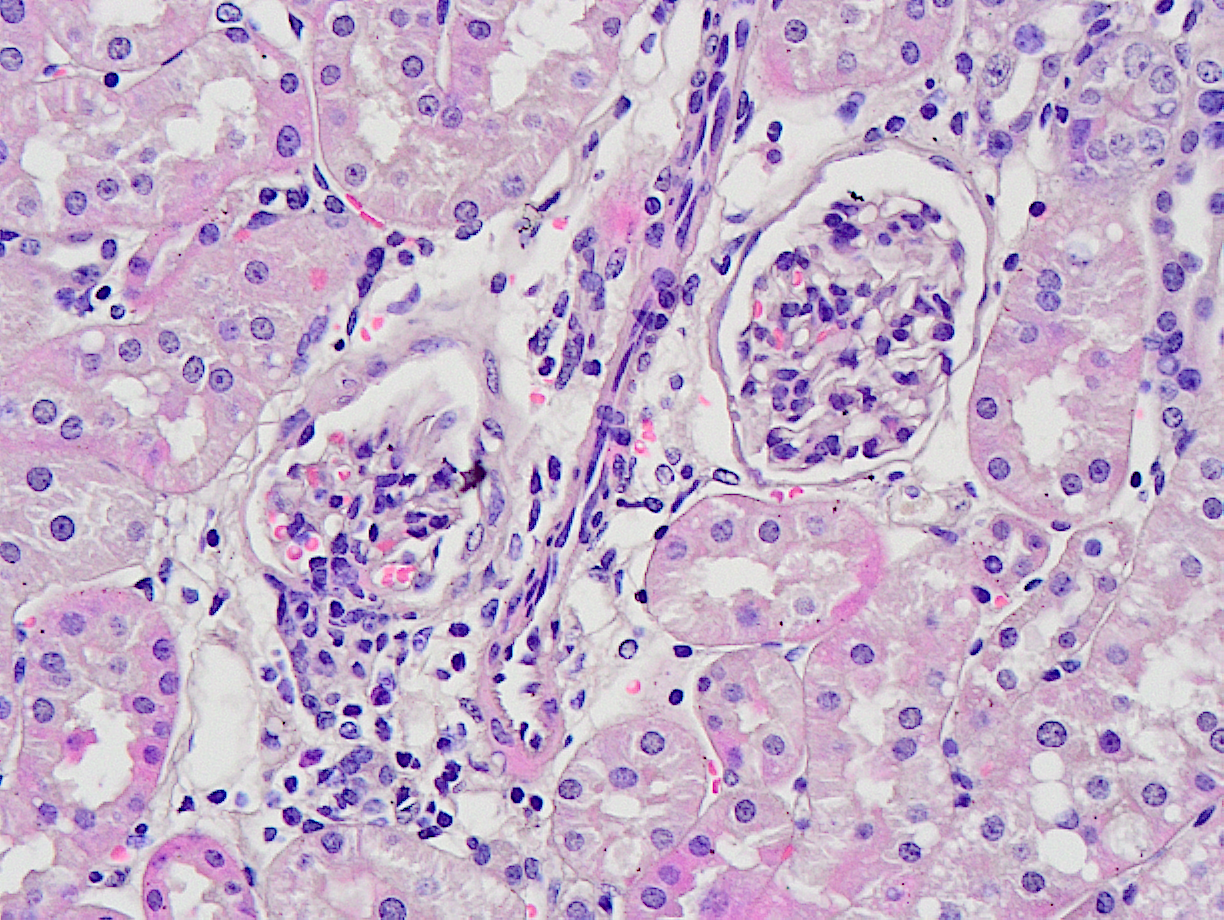

Supplement: Supplementary file 5 [file DataSheet2.ZIP › the original Images,fig1/H&E/DM.tif]

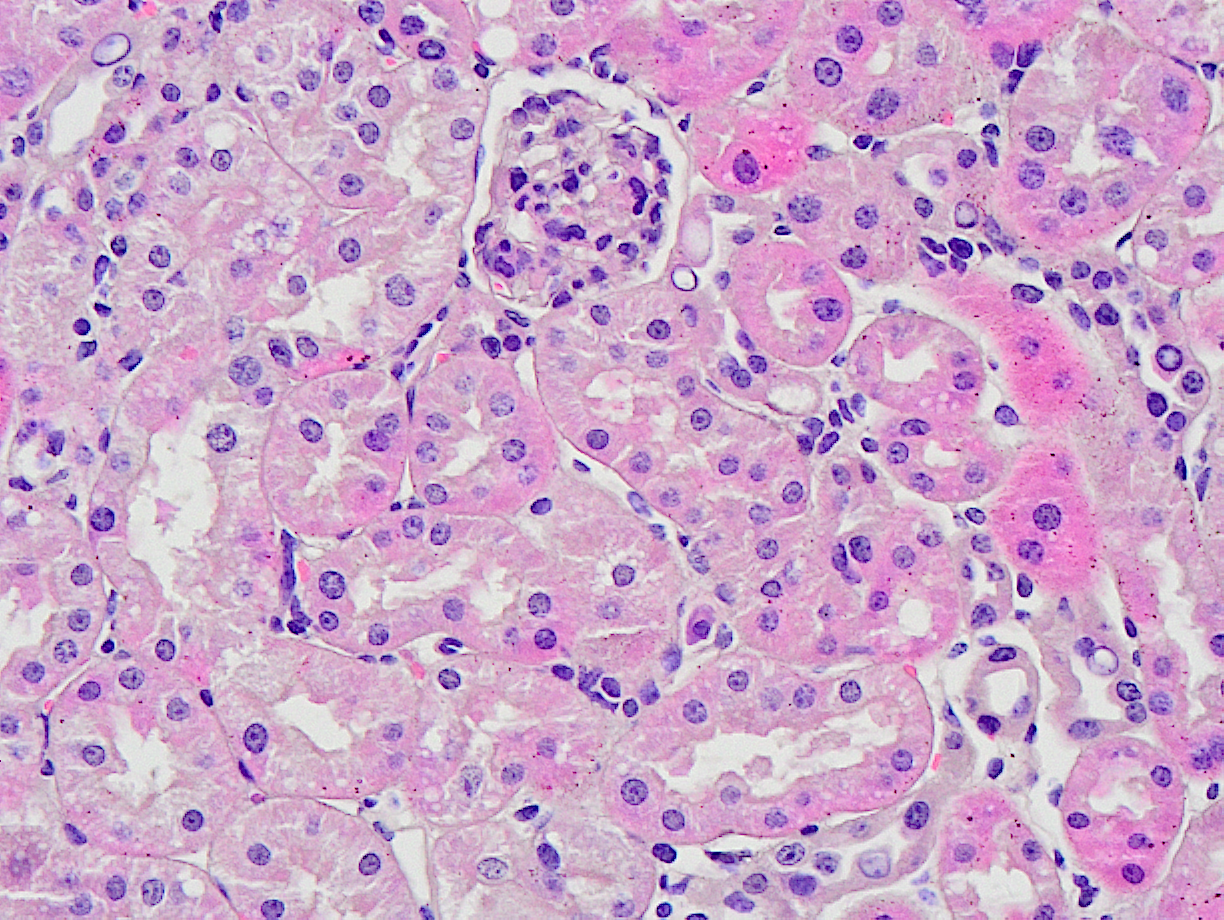

Supplement: Supplementary file 5 [file DataSheet2.ZIP › the original Images,fig1/H&E/ATO.tif]

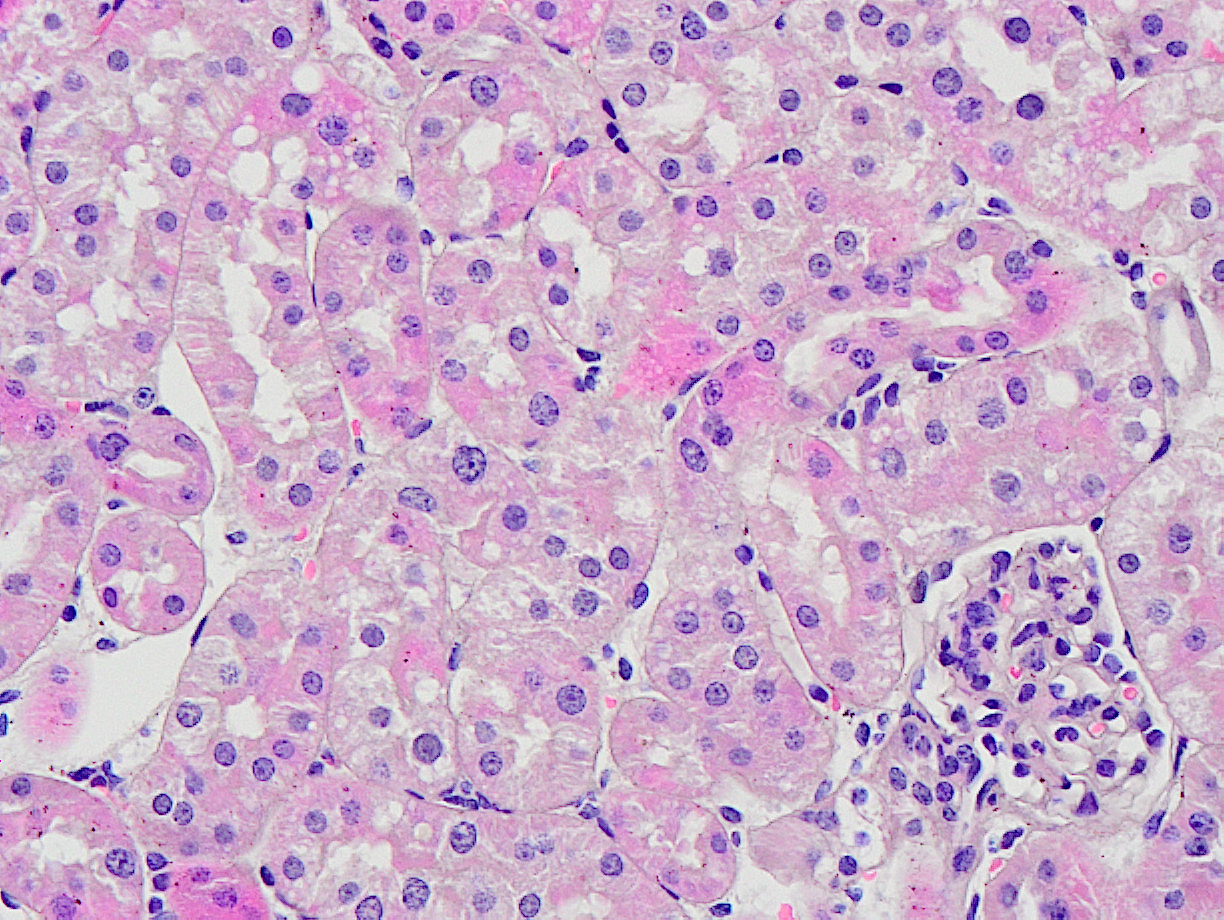

Supplement: Supplementary file 5 [file DataSheet2.ZIP › the original Images,fig1/H&E/NC1 400.tif]

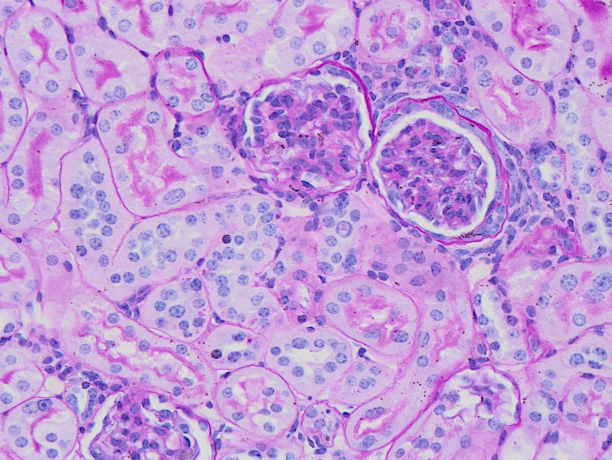

Supplement: Supplementary file 5 [file DataSheet2.ZIP › the original Images,fig1/PAS/DM.tif]

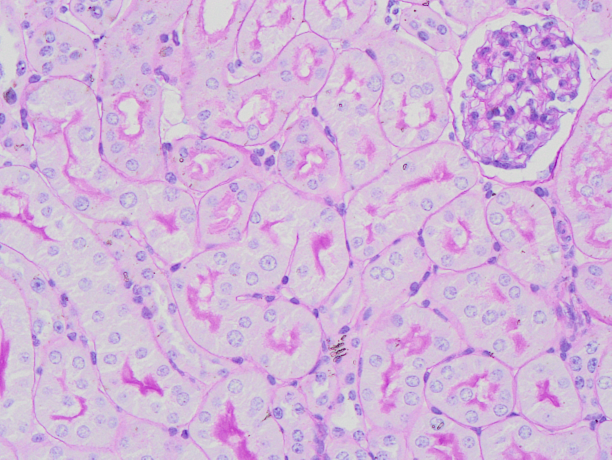

Supplement: Supplementary file 5 [file DataSheet2.ZIP › the original Images,fig1/PAS/ATO.tif]

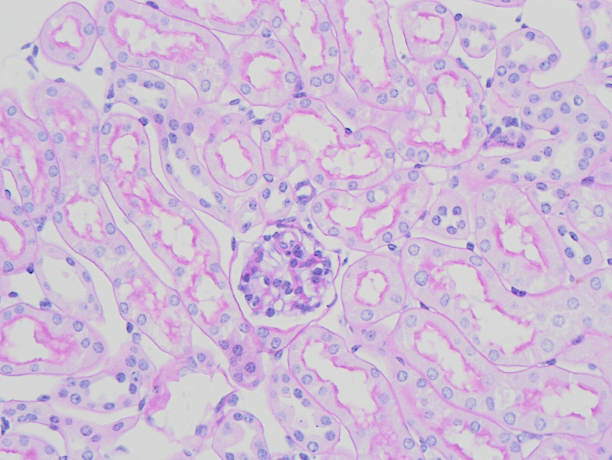

Supplement: Supplementary file 5 [file DataSheet2.ZIP › the original Images,fig1/PAS/NC.tif]

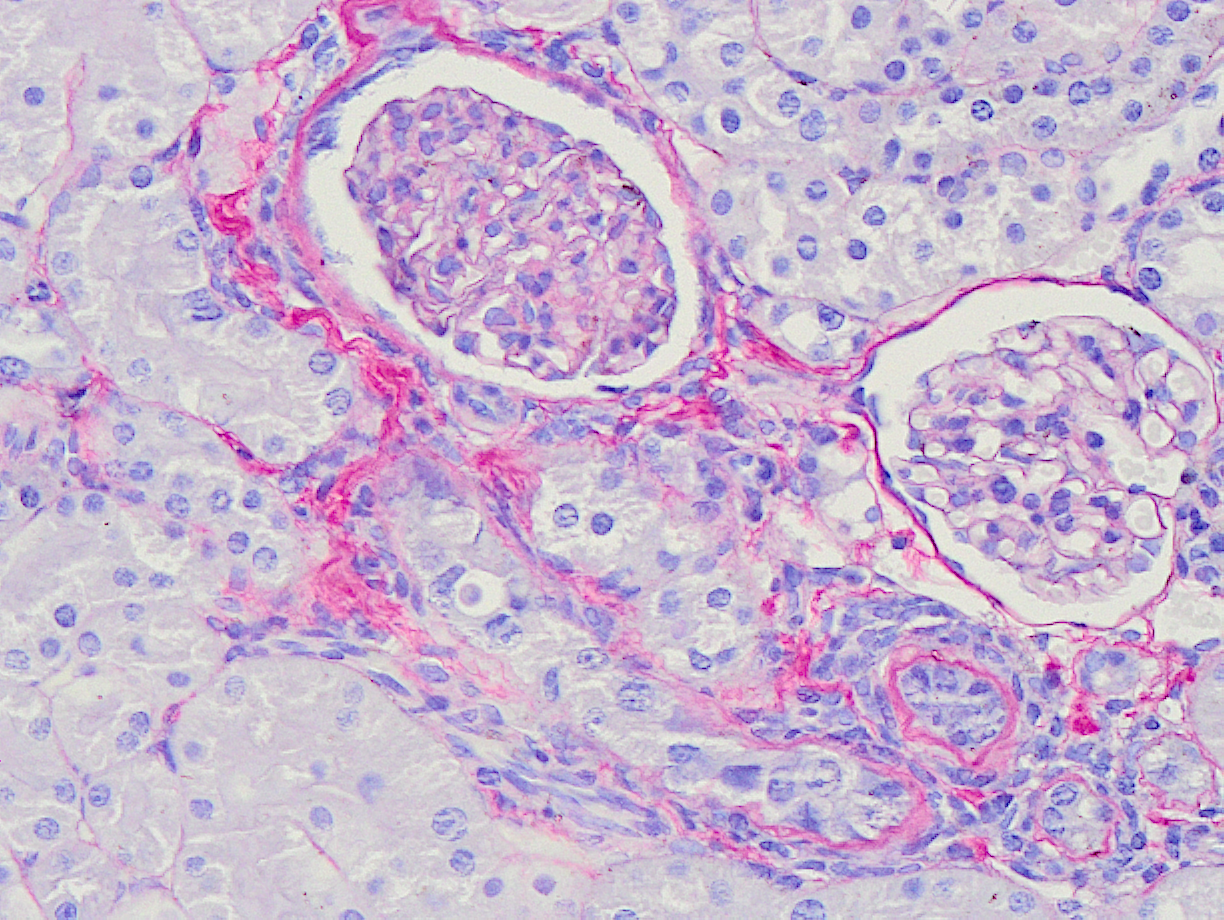

Supplement: Supplementary file 5 [file DataSheet2.ZIP › the original Images,fig1/Sirius Red/DM 400.tif]

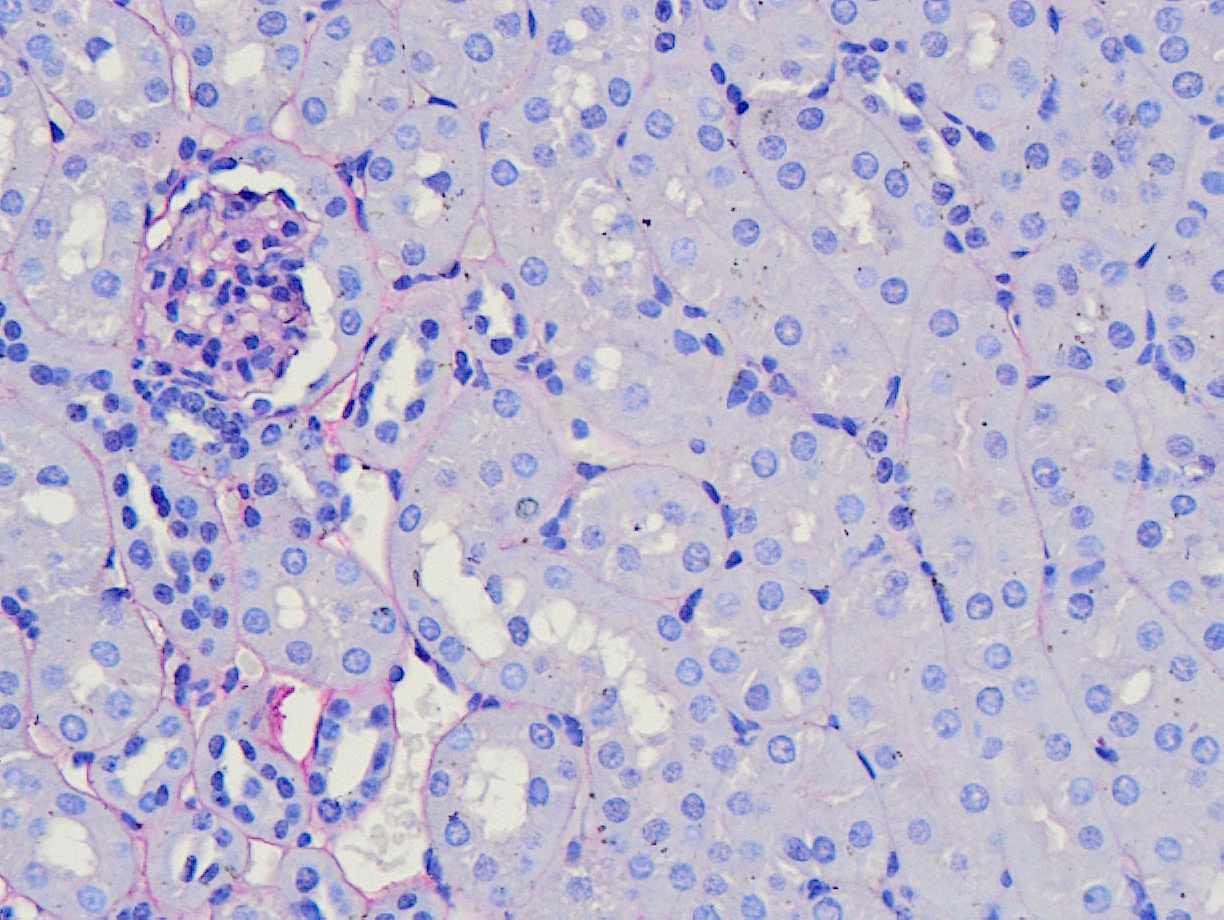

Supplement: Supplementary file 5 [file DataSheet2.ZIP › the original Images,fig1/Sirius Red/ATO 1 400.tif]

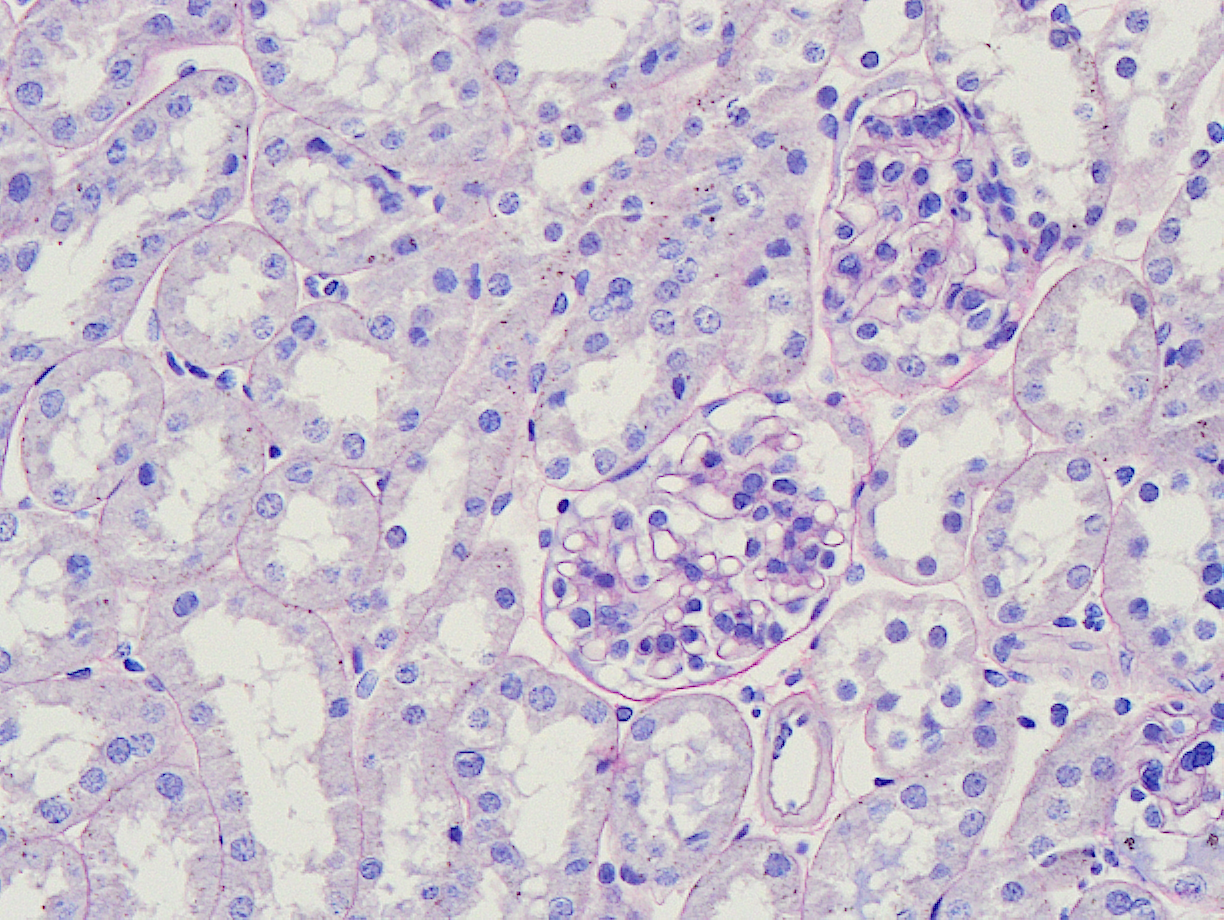

Supplement: Supplementary file 5 [file DataSheet2.ZIP › the original Images,fig1/Sirius Red/NC 400.tif]
