# Supplementary material for: Atorvastatin Restores PPARα Inhibition of Lipid Metabolism Disorders by Downregulating miR-21 Expression to Improve Mitochondrial Function and Alleviate Diabetic Nephropathy Progression
Source: Front Pharmacol. 2022 Feb 11;13:819787. doi: 10.3389/fphar.2022.819787 (PMC8874267; doi:10.3389/fphar.2022.819787)
Supplement: Supplementary file 2 [file DataSheet4.ZIP › Supplementary experimental original data/Approvel Form for Animal Experiment Ethics Group of Guizhou Medical university.pdf]

贵州医科大学
GUIZHOU MEDICAL UNIVERSITY

动物实验伦理审查表

Animal Experimental Ethical Inspection Form of Guizhou Medical University

编号 (No)： [illegible]1602230

申请人填写的信息 (Related information filled by applicant)

申请单位<br>Name of organization	贵州医科大学<br>Guizhou Medical University		
项目经费来源<br>Funding source	贵阳市科技计划<br>Guiyang Science and Technology Plan		
申请人<br>Applicant	郭兵<br>Bing Guo	联系电话<br>Telephone	13908518950
项目名称<br>Experiment title	阿托伐他汀通过调节三种 microRNA 治疗糖尿病肾病肾间质纤维化的研究		
申请日期<br>Application date	2016 年 11 月 13 日<br>November 13, 2016		
拟实验时间<br>Experiment date	2016 年 12 月 1 日至 2019 年 12 月 1 日<br>December 1, 2016 to December 1, 2019		
使用动物情况 品种品系<br>Species of strain	小鼠，C57BL/6	等级<br>Grade	普通级<br>General grade
使用动物情况 实验设施合格证编号<br>Reg. No. of Experimental Facilities certification	SYXK(黔) 2018-0001	规格<br>Specifications	4 – 6 weeks,<br>18 – 22g, males

实验要点：包括实验目的、实验方法、观测指标、实验结束后处死动物的方法等
Outline of experiments, including aim of experiment, experimental methods, observational index, executing animal method, et al.

实验目的：本研究拟以 I 型糖尿病小鼠模型、高糖培养的小鼠肾小管上皮细胞和大鼠原代肾小管上皮细胞模型为研究对象，探讨阿托伐他汀干预治疗对小鼠肾脏和肾小管上皮细胞纤维化的影响，以及对 miR-21、miR-22、和 miR-124 表达的影响。本课题的完成将进一步揭示阿托伐他汀的作用机制，为其更好的用于临床提供坚实的理论依据和实验支持。

Aim of experiment: This study intends to investigate the effects of atorvastatin intervention treatment on mouse kidney and renal tubular epithelial cell fibrosis, and the effects on miR-21, miR-22, and miR-124 expression, using a mouse model of type I diabetes, a mouse renal tubular epithelial cell cultured with high glucose, and a rat primary renal tubular epithelial cell model. The completion of this project will further reveal the mechanism of action of atorvastatin and provide a solid theoretical basis and experimental support for its better clinical use.

实验方法：将动物随机分为正常对照组(NC)组(n=6)和 DM 组(n=12)。DM 组小鼠腹腔注射链脲佐菌素(STZ, Sigma) 55 mg/kg;NC 组小鼠连续注射等量的 ph4.5 无菌柠檬酸-柠檬酸钠缓冲液(溶菌酶)5 天。治疗后 72h 测定小鼠空腹血糖，血糖值≥16.7 mmol/L 表示 DM 小鼠造模成功。喂食 5 周后，将糖尿病小鼠随机分为糖尿病组(n=6)和 Ato 组(DM+Ato)(n=6)。Ato 组给予阿托伐他汀 20mg /(kg · d)(中国辉瑞公司)，连续 4 周。NC 组和 DM 组小鼠灌胃羧甲基纤维素 4 周。第 9 周取小鼠肾脏。在安乐死前 24 小时内采集尿液样本并测量其体积。所有小鼠在祭祀前禁食 6 h。从股动脉采集血液标本，离心制备血清，保存在-20 ˚

	C 用于生化评估．取下两个肾脏，其中一个保存在- 80 ° C (RNA 和蛋白制备)，另一个用 4%的福尔马林固定以进行组织学和免疫组化评估．<br>Experimental methods: The animals were randomly divided into the normal control(NC)group(n=6) and the DM group (n=12).The mice of the DM group were intraperitoneally injected with 55 mg/kg streptozotocin (STZ, Sigma); mice in the NC group were injected with the same amount of pH 4.5 sterile citric acid-sodium citrate buffer (lysozyme) for 5 consecutive days.Fasting blood glucose levels in mice were assessed at 72h after treatment, and values ≥ 16.7 mmol/L indicated that DM mice were successfully modeled.After 5 weeks of feeding, the diabetic mice were randomized into diabetic group(n=6) and Ato group (DM+Ato)(n=6). Atorvastatin 20mg /(kg · d)(Pfizer,China) was given to the Ato group for four weeks.NC group and DM group were intragastrically administered with carboxymethylcellulose for 4 weeks.The mouse kidneys were collected at 9th week. Urine samples were obtained and measured for volume in the 24 - hr period preceding euthanasia. All mice were fasted for 6 h prior to sacrifice. Blood specimens were collected from the femoral artery and centrifuged for preparing serum, kept at −20 ° C for biochemical assessment. Both kidneys were removed, with one stored at −80 ° C (RNA and protein preparations) and the other submitted to fixation with 4% formalin for histological and immunohistochemical evaluations.<br>观测指标：生化方法测血糖和尿蛋白，记录 24h 尿量，计算 24h 尿蛋白量; HE、PAS 和天狼星红染色后，光镜观察肾组织病理变化;免疫组织化学及免疫印迹检测各目的蛋白在各组肾组织中的表达部位和表达量;Real-time PCR 检测 miR-21 及 PPAR-α mRNA 在肾组织中的表达；以高糖培养的肾小管上皮细胞（NRK52E）为研究对象，敲减 miR-21 的表达或给予 PPAR-α 特异性激动剂非诺贝特干预；流式细胞术检测线粒体膜电位、ROS 产生情况；双荧光素酶报告实验验证 miR-21 对 PPAR-α 转录调控作用．<br>Observations: biochemical methods to measure blood glucose and urine protein, record 24h urine volume, and calculate 24h urine protein volume; light microscopic observation of renal histopathological changes after HE, PAS, and Sirius red staining; immunohistochemistry and immunoblotting to detect the expression sites and expression of each target protein in each group of renal tissues; Real-time PCR to detect miR-21 and PPAR-α mRNA in renal The expression of miR-21 and PPAR-α mRNA in kidney tissues was examined by Real-time PCR; the expression of miR-21 was knocked down or fenofibrate, a PPAR-α -specific agonist, was administered to high glucose cultured renal tubular epithelial cells (NRK52E); the mitochondrial membrane potential and ROS production were detected by flow cytometry; the dual-luciferase reporter assay was performed to verify the role of miR-21 on the transcriptional regulation of PPAR-α.<br>实验结束后处死动物的方法：麻醉后处死．<br>Method of execution of animals at the end of the experiment: anesthesia followed by execution.

（请翻看背面）

<table>
<tr><td>申请者声明
Announcement of applicant</td><td colspan="2">我将自觉遵守实验动物福利伦理原则，随时接受实验动物伦理委员会的监督与检查，如违反规定，自愿接受处罚。
I will abide by the rules of animal experimental ethics, accept the supervision and inspection of the animal experimental ethics committee, and accept the punishment in case of any infringement.)

申请者签名：[illegible]
2016年11月13日</td></tr>
<tr><td>审查依据
Inspection contents</td><td colspan="2">1．该项目是否必须用实验动物进行实验，即能否用计算机模拟、细胞培养等非生命方法替代动物或用低等动物替代高等动物进行实验(Does laboratory animal must be used in the project? Could other methods such as computer simulation．cell culture or using the low-grade animal instead of the high – grade animal?)
2．表中所填申请人资格和所用动物的品种品系、质量等级、规格是否合适，能否通过改良设计方案或用高质量的动物来减少所用动物的数量(Are the qualification of applicant, species or strain，grade and specifications of animals suitable? Could the quantity of animals be reduced by improving the study design or using high quality animals?)
3．能否通过改进实验方法、调整实验观测指标、改良处死动物的方法，来优化实验方案、善待动物(Could the study design and animal treatment be refined by ameliorating experimental method，adjusting observational index．executing animal method?)</td></tr>
<tr><td rowspan="3">审查结果
Results of inspection</td><td>课题负责人意见
Project director attitude</td><td>同意　　签名：[illegible]</td></tr>
<tr><td>实验动物伦理委员会委员意见
Members attitude of the Animal Care Welfare Committee</td><td>同意　　签名：杨红宇</td></tr>
<tr><td>实验动物伦理委员会
Attitude of the Animal Care Welfare Committee</td><td>贵州医科大学
GuiZhou Medical University
实验动物伦理委员会
the Animal Care Welfare Committee
签章
2016年11月25日</td></tr>
<tr><td colspan="3">备注：
Remark</td></tr>
</table>

说明：

1.申请表审核结束后，一式2份递交到贵州医科大学实验动物中心盖章。
2.课题负责人、执行人及合作单位负责人均需在申请者签字栏签字。
3.需在外单位完成课题的，请同时填写校外实验动物设施使用证明。
4.表格签名处必须手写。
5.要求写明项目的意义、必要性、项目中有关实验动物的用途、饲养管理或实验处置方法、预期出现对动物的伤害、处死动物的方法、项目进行中涉及动物福利。
