# Supplementary material for: Atorvastatin Restores PPARα Inhibition of Lipid Metabolism Disorders by Downregulating miR-21 Expression to Improve Mitochondrial Function and Alleviate Diabetic Nephropathy Progression
Source: Front Pharmacol. 2022 Feb 11;13:819787. doi: 10.3389/fphar.2022.819787 (PMC8874267; doi:10.3389/fphar.2022.819787)
Supplement: Supplementary file 3 [file DataSheet1.ZIP › flow cytometry data πÇüSPSS statisticsπÇügels/the entire original gels.pptx]

## Slide 1
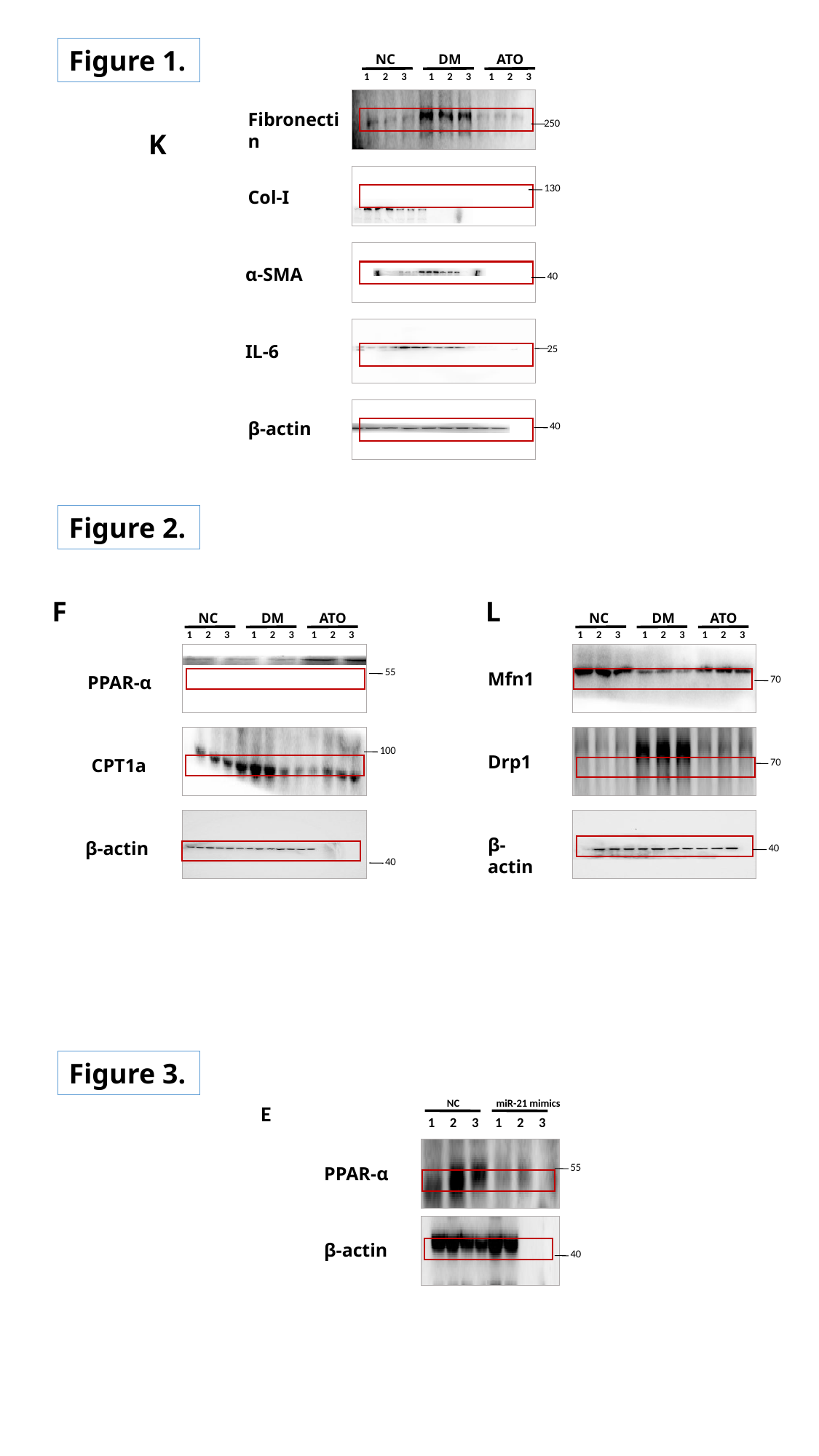

Figure 1.
NC
DM
ATO
1
2
3
1
2
3
1
2
3
Fibronectin
250
K
130
Col-I
α-SMA
40
IL-6
25
β-actin
40
Figure 2.
F
L
NC
DM
ATO
1
2
3
1
2
3
1
2
3
NC
DM
ATO
1
2
3
1
2
3
1
2
3
55
Mfn1
PPAR-α
70
100
Drp1
CPT1a
70
β-actin
β-actin
40
40
Figure 3.
NC
miR-21 mimics
1
2
3
1
2
3
E
55
PPAR-α
β-actin
40

## Slide 2
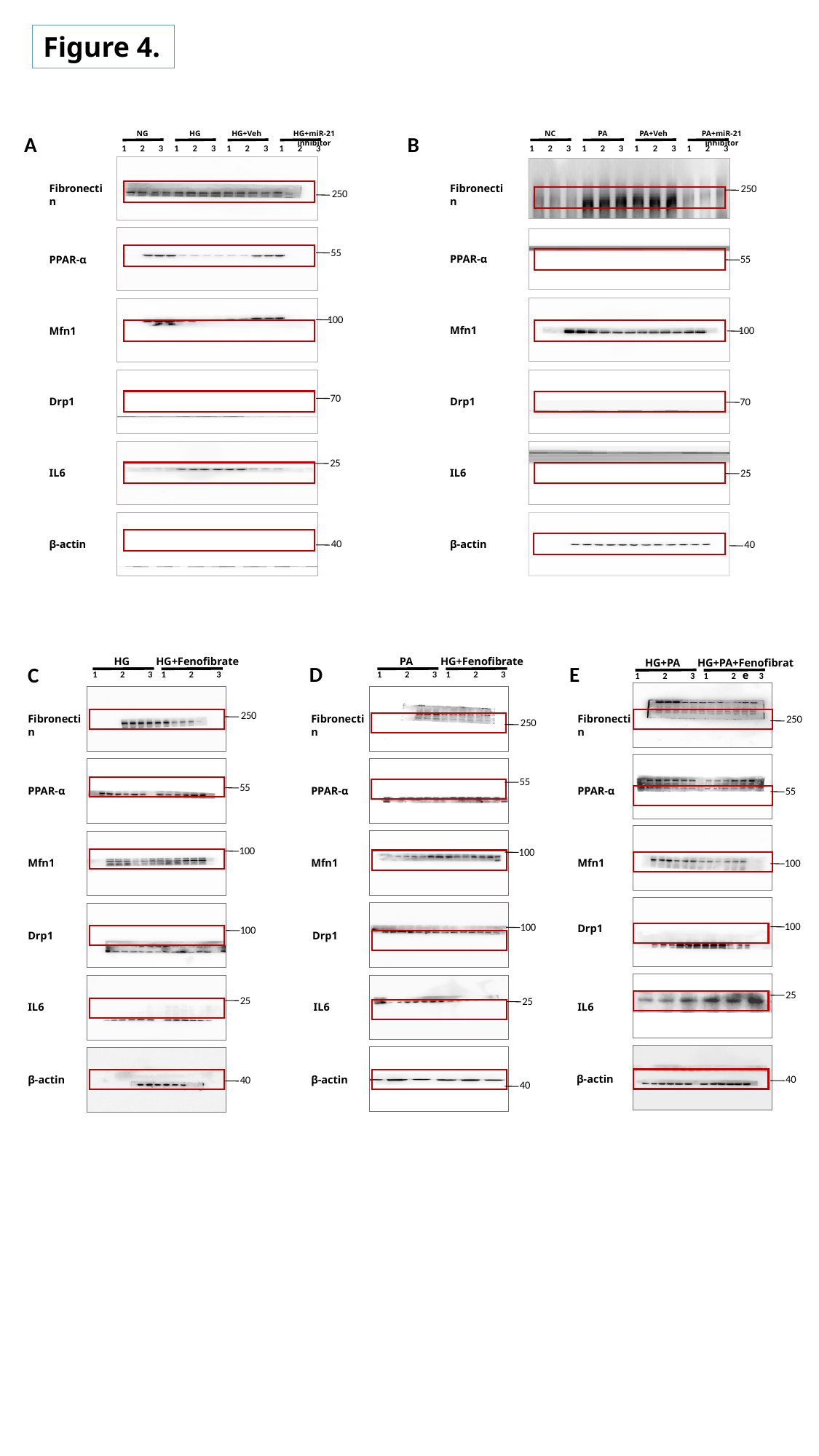

Figure 4.
NC
PA
PA+Veh
PA+miR-21 inhibitor
1
2
3
1
2
3
1
2
3
1
2
3
NG
HG
HG+Veh
HG+miR-21 inhibitor
1
2
3
1
2
3
1
2
3
1
2
3
A
B
250
Fibronectin
Fibronectin
250
55
PPAR-α
55
PPAR-α
100
Mfn1
100
Mfn1
70
Drp1
70
Drp1
25
IL6
25
IL6
40
β-actin
β-actin
40
HG
1
2
3
HG+Fenofibrate
1
2
3
PA
1
2
3
HG+Fenofibrate
1
2
3
HG+PA
1
2
3
HG+PA+Fenofibrate
1
2
3
D
E
C
250
Fibronectin
Fibronectin
Fibronectin
250
250
55
55
PPAR-α
55
PPAR-α
PPAR-α
100
100
Mfn1
100
Mfn1
Mfn1
100
100
Drp1
100
Drp1
Drp1
25
25
25
IL6
IL6
IL6
β-actin
40
β-actin
40
β-actin
40

## Slide 3
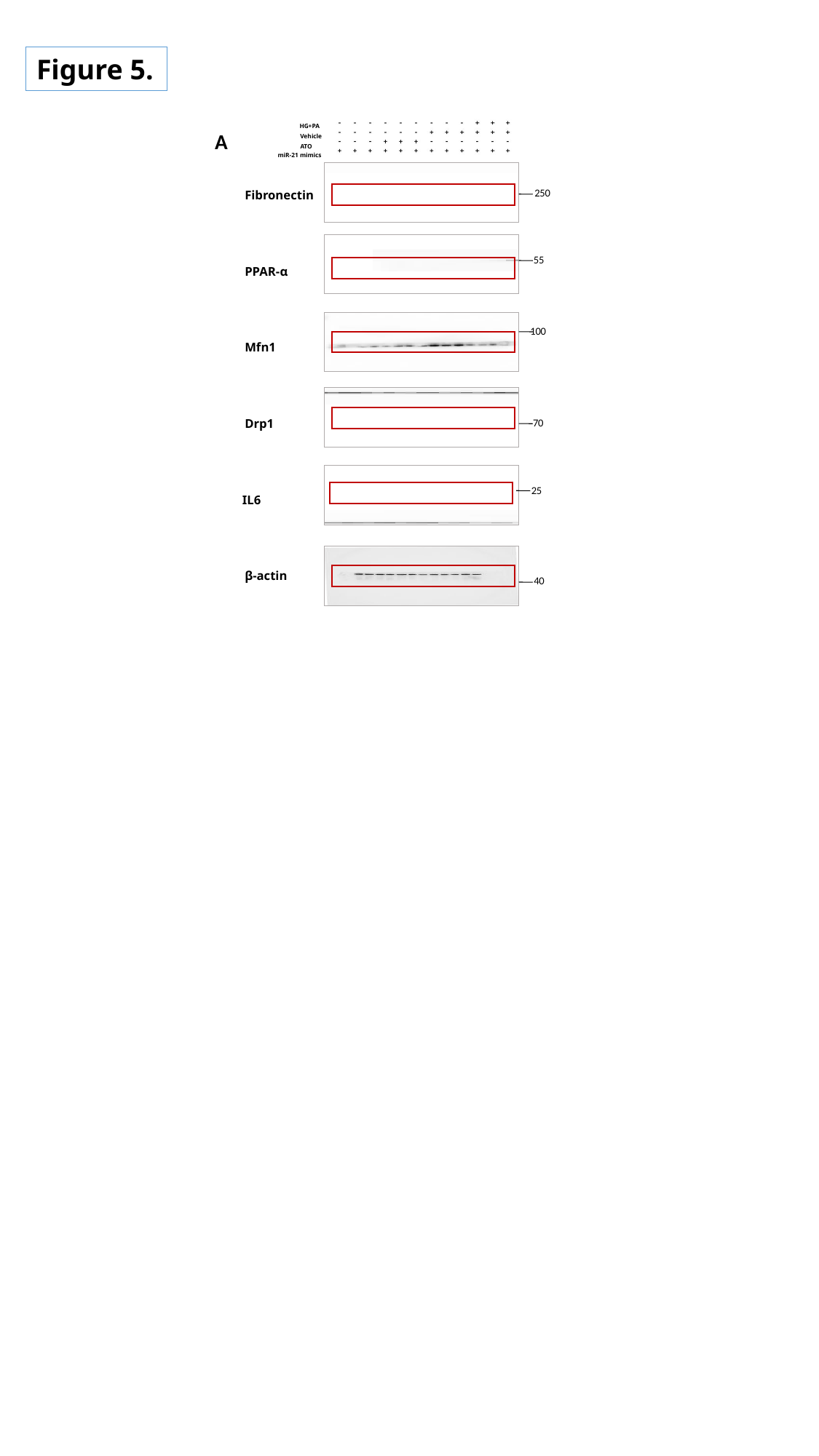

Figure 5.
HG+PA
Vehicle
ATO
miR-21 mimics
A
250
Fibronectin
55
PPAR-α
100
Mfn1
Drp1
70
25
IL6
β-actin
40
